# Heterometric expression of an LBD gene via LBD-TCP assembly regulates floral organ size and fruit weight in Physalis

**DOI:** 10.1093/hr/uhaf211

**Published:** 2025-08-11

**Authors:** Qiaoru Li, Lanfeng Wu, Jing Li, Qianhui Cao, Pichang Gong, Li Wang, Nan Xu, Chaoying He

**Affiliations:** State Key Laboratory of Plant Diversity and Specialty Crops/State Key Laboratory of Systematic and Evolutionary Botany, Institute of Botany, Chinese Academy of Sciences, Nanxincun 20, Xiangshan, Beijing 100093, China; China National Botanical Garden, Beijing 100093, China; University of Chinese Academy of Sciences, Yuquan Road 19, Beijing 100049, China; State Key Laboratory of Plant Diversity and Specialty Crops/State Key Laboratory of Systematic and Evolutionary Botany, Institute of Botany, Chinese Academy of Sciences, Nanxincun 20, Xiangshan, Beijing 100093, China; China National Botanical Garden, Beijing 100093, China; University of Chinese Academy of Sciences, Yuquan Road 19, Beijing 100049, China; State Key Laboratory of Plant Diversity and Specialty Crops/State Key Laboratory of Systematic and Evolutionary Botany, Institute of Botany, Chinese Academy of Sciences, Nanxincun 20, Xiangshan, Beijing 100093, China; University of Chinese Academy of Sciences, Yuquan Road 19, Beijing 100049, China; State Key Laboratory of Plant Diversity and Specialty Crops/State Key Laboratory of Systematic and Evolutionary Botany, Institute of Botany, Chinese Academy of Sciences, Nanxincun 20, Xiangshan, Beijing 100093, China; China National Botanical Garden, Beijing 100093, China; University of Chinese Academy of Sciences, Yuquan Road 19, Beijing 100049, China; State Key Laboratory of Plant Diversity and Specialty Crops/State Key Laboratory of Systematic and Evolutionary Botany, Institute of Botany, Chinese Academy of Sciences, Nanxincun 20, Xiangshan, Beijing 100093, China; China National Botanical Garden, Beijing 100093, China; State Key Laboratory of Plant Diversity and Specialty Crops/State Key Laboratory of Systematic and Evolutionary Botany, Institute of Botany, Chinese Academy of Sciences, Nanxincun 20, Xiangshan, Beijing 100093, China; China National Botanical Garden, Beijing 100093, China; State Key Laboratory of Plant Diversity and Specialty Crops/State Key Laboratory of Systematic and Evolutionary Botany, Institute of Botany, Chinese Academy of Sciences, Nanxincun 20, Xiangshan, Beijing 100093, China; China National Botanical Garden, Beijing 100093, China; University of Chinese Academy of Sciences, Yuquan Road 19, Beijing 100049, China; State Key Laboratory of Plant Diversity and Specialty Crops/State Key Laboratory of Systematic and Evolutionary Botany, Institute of Botany, Chinese Academy of Sciences, Nanxincun 20, Xiangshan, Beijing 100093, China; China National Botanical Garden, Beijing 100093, China; University of Chinese Academy of Sciences, Yuquan Road 19, Beijing 100049, China

## Abstract

Plant LATERAL ORGAN BOUNDARIES DOMAIN (LBD) family is crucial for defining organ boundaries and participates in various developmental processes, but its role in fruit weight has rarely been elucidated. Here, we characterized an LBD gene, *Physalis organ size 3* (*POS3*), in *Physalis floridana*, designated as *PfPOS3*. This gene exhibited high expression levels in floral meristems, carpels, and developing seeds and fruits when compared to *Solanum pimpinellifolium* and *Solanum lycopersicum*. The floral organ size, seed weight, and mature fruit weight were significantly reduced in *PfPOS3* knockdown and knockout plants. Consistent with overexpression analyses, *PfPOS3* promoted cell size and inhibited cell division during berry development. Moreover, overexpression of *PfPOS3* and *SlPOS3* shared identical phenotypic variation in transgenic Physalis plants. Both PfPOS3 and SlPOS3 interacted with Teosinte branched1/Cycloidea/Proliferating cell factor 15 (TCP15) and TCP18, and the POS3-TCP modules directly regulated the expression of *Cyclin D1;1* (*CYCD1;1*) and *CYCB1;1*. Overall, POS3 may have the capability to orchestrate cell number and cell size, thus regulating fruit weight variation within Solanaceae. However, a significant reduction in the expression of *SlPOS3* may result in a pronounced weakening or complete loss of this function within *Solanum*. Our findings shed new light on the reproductive organ size control, the developmental evolution of fruit morphology, and the breeding of Physalis crops.

## Introduction

The plant-specific transcription factor (TF) LATERAL ORGAN BOUNDARIES DOMAIN (LBD) family plays an essential role in defining organ boundaries and is involved in multiple aspects of plant development, including lateral root formation, shoot-borne roots, embryogenesis, callus formation, leaf patterning, flowering time, flower and inflorescence development, pollen development, vascular patterning, anthocyanin, meristem programming, and the regulation of secondary growth and nitrogen metabolism [1–7]. In *Arabidopsis*, *AtLBD18* is expressed in lateral root primordia and emerging lateral roots [8]. Mutant *atlbd18* plants exhibit a reduced number of lateral roots compared to wild-type (WT) plants. *AtLBD18* overexpression in the *Arabidopsis auxin response factor7/19* (*arf7 arf19*) mutant rescues the phenotypic variation in reduced lateral roots [8]. Further studies have shown that ARF7 and ARF19 can directly bind to the AuxRE element in the *AtLBD18* promoter, while AtLBD18 binds to the *ARF19* promoter to regulate its expression *in vivo* [9, 10]. AtLBD18 also activates *EXPANSIN14* by directly binding to the *EXPANSIN14* promoter *in vivo* to promote lateral root emergence in *Arabidopsis* [11]. In tomato, the LBD gene *SHOOT BORNE ROOTLESS* (*SlSBRL*) regulates root initiation, enabling the plant’s complex developmental response to varied environments [7], and a few LBD genes are associated with tomato fruit development thus far. *SlLOB1* upregulates a suite of cell wall-associated genes during the late maturation and ripening of fruit [12], while LBD genes *SlAS2* (*ASYMMETRIC LEAVES 2*) and *SlAS2L* (*SlAS2-LIKE*) have redundant and pleiotropic functions in tomato fruit development by regulating cell division and cell differentiation genes [13]. *SlLBD40* co-expressing with *SlMYC2* stimulates the activation of *SlEXPA5*, leading to an increase in fruit size [14]. But the function of LBD genes in fruit development is largely unknown.

The Solanaceae family contains more than 3000 species, with rich diversity in fruit types and morphologies [15, 16]. Fruit weight, as a domesticated and improved trait, is an important factor in determining the commercial value of solanaceous crops [17]. Tomato (*Solanum lycopersicum*) is a commercial crop grown worldwide that plays an important role in fruit-based vegetable supply. Many quantitative trait loci (QTLs) in controlling the weight variation of tomato fruit have been discovered [18], but only a few important QTLs and genes have been cloned thus far, including *FRUIT WEIGHT 2*.*2* (*FW2*.*2*), *FRUIT WEIGHT 3*.*2* (*FW3*.*2*), *FASCIATED* (*FAS*), *LOCULE NUMBER* (*LC*), and *EXCESSIVE NUMBER OF FLORAL ORGANS* (*SlENO*) [19–23]. Physalis, the second-largest genus in the Solanaceae family, has a morphological novelty known as the ‘Chinese lantern’ or inflated calyx syndrome (ICS) [16, 24]. Some Physalis species produce berries used in affinal drugs and diets [25], but the small berry size is a major limiting factor for high fruit yield. A few genes regulating fruit size have been characterized in this genus. The expression of *PHYSALIS ORGAN SIZE 1* (*POS1*), encoding a tandem APETALA2-cytokinin response factor, has a significantly positive correlation with the berry size of *P*. *philadelphica* [26], while only POS1 from Physalis and its Capsicum ortholog (CaPOS1) are co-opted for fruit size control via a lineage-specific arginine in these two genera [27]. *Physalis floridana CELL NUMBER REGULATOR 1* (*PfCNR1*), also designated *POS2*, an orthologous gene of tomato *FW2*.*2*, regulates berry size by interacting with an MADS-domain TF PFAG2 [28]. PFAG2 selectively binds to the CArG-box in the *PfCYCD2;1* promoter and represses *PfCYCD2;1* expression to participate in cell division [28]. Editing the tomato *CLV1* orthologous gene, encoding one of several redundant leucine-rich receptors of the FAS (CLV3) peptide in *P*. *pruinosa*, also affects fruit size by increasing the locule number [29]. This suggests the uniqueness and conservation of the genetic basis regulating fruit weight in Solanum and Physalis; however, it remains largely unknown.

Forward genetics can precisely target the causative gene for trait variation; however, QTL analysis for cloning genes regulating fruit size in Physalis is hampered due to the difficulty in interspecific hybridization. Transcriptomic comparisons at the flower–fruit transition between *P. floridana* and *S. pimpinellifolium* may reveal large-scale differentially expressed genes (DEGs) that serve as an arsenal of candidate genes for solanaceous fruit development [30]. Our preliminary functional analysis using the virus-induced gene silencing (VIGS) approach showed that *P5* may have a putative function in regulating floral organ size and fruit weight in *P*. *floridana* [30], but the exact roles and underlying mechanisms remain unclear. In this study, *P5* was designated as *PHYSALIS ORGAN SIZE 3* (*POS3*), which encoded a putative LBD TF. We confirmed the roles of *POS3* in regulating fruit development via gene editing, RNAi, and overexpression approaches in *P. floridana* and clarified its positively regulatory roles in reproductive organ size, demonstrating its involvement in cell cycle control. We also found that *P. floridana* POS3 (PfPOS3) interacted with two TCP proteins and bound to the promoters of *Cyclin* (*CYC*) genes *CYCB1;1* and *CYCD1;1*, thus regulating their expression. However, *POS3-*like mRNA was highly expressed in floral and fruit development in *P. floridana* compared to its orthologs in *S. lycopersicum* and *S. pimpinellifolium*. We demonstrated that POS3-TCP-CYC pathways participated in Physalis reproductive organ development, thus providing new insights into the evolution of flower and fruit size in Solanaceae.

**Figure 1:**
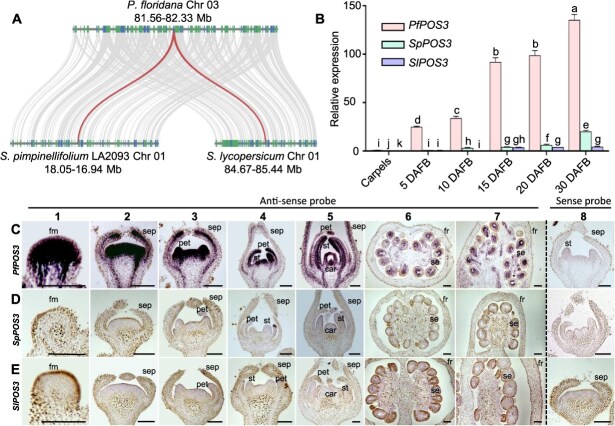
Synteny and expression pattern of *POS3-*like. (**A**) Synteny of *POS3-*like in *P. floridana*, *S. pimpinellifolium*, and *S. lycopersicum*. The red linear region represents *POS3-*like, and the gray linear region represents genes around *POS3-*like on the syntenic regions. (**B**) Tissue-specific mRNA accumulation in the three indicated species. Total RNA from the indicated tissues/organs was subjected to qRT-PCR analysis. *PfPOS3* expression in the carpel was set at 1.0. Mean and standard deviation (SD) are presented (*n* = 3). *PfActin*, *SpActin*, or *SlActin* were used as an internal control. DAFB, berry of days after fertilization. Different lowercase letters represent significant difference (*P* < 0.05) in two-tailed Student’s *t*-test. (**C**) *In situ* hybridization detection of *PfPOS3* transcripts in floral organs and fruit 0 DAF in *P*. *floridana*. (**D**) *In situ* hybridization detection of *SpPOS3* transcripts in floral organs and fruit 0 DAF in *S*. *pimpinellifolium*. (**E**) *In situ* hybridization detection of *SlPOS3* transcripts in floral organs and fruit 0 DAF in *S*. *lycopersicum*. In **C**–**E**, 1–7 represents the stages of floral primordia, sepal primordia, petal primordia, stamen primordia, carpel primordia, fruit 0 DAF (transverse cutting), and fruit 0 DAF (longitudinal cutting), respectively. Eight, respectively, represents the stages of stamen primordia (**C**), petal primordia (**D**), and calyx primordia (**E**). Different tissues were hybridized by antisense (1–7) and sense (8) probes for each indicated gene. fm, floral meristem; sep, sepal primordia; pet, petal primordia; st, stamen primordia; car, carpel primordia; fr, fruit; se, seed. Bars = 100 μm.

**Figure 2:**
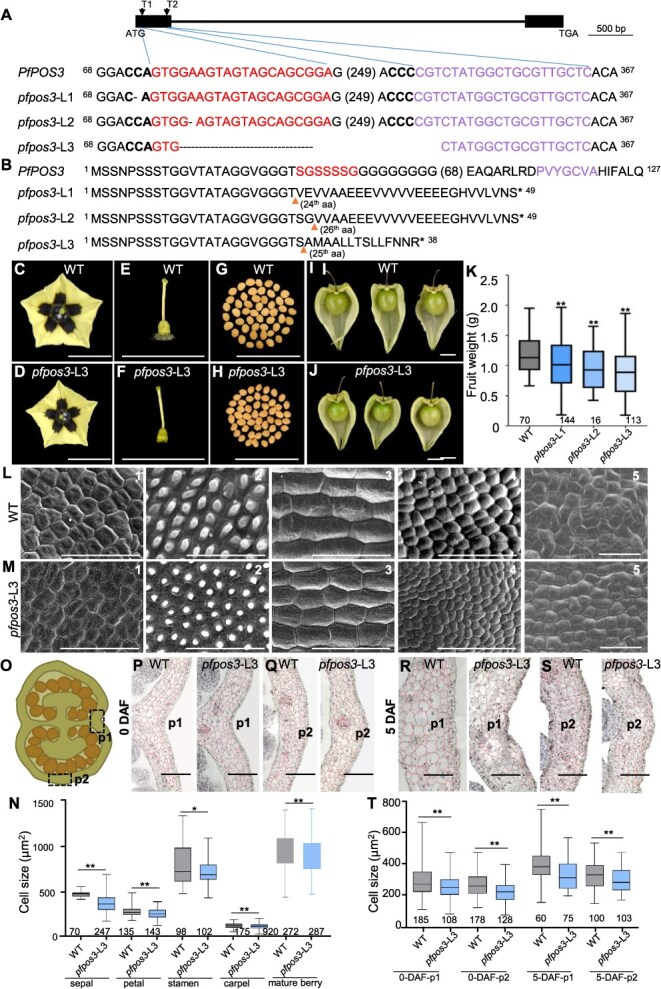
Generation and analyses of *pfpos3*-edited *P*. *floridana* plants. (**A**) Gene-editing mode in three independent *pfpos3*-edited lines. T1 and T2 represent the position of two single-guide RNAs (sgRNAs) on the first exon of the *PfPOS3* genomic structure. Sequences of three different *pfpos3* lines are shown compared to *PfPOS3*. The sgRNA targets T1 and T2 are indicated in red and purple, respectively. The protospacer-adjacent motifs (PAM) are indicated in bold font. Deletions are indicated by dashes. (**B**) Putative PfPOS3 protein alterations in *pfpos3*-edited lines. Relative to PfPOS3, editing caused premature stop codons in the three *pfpos3*-edited lines. The orange arrowheads indicate the frameshift sites in the putative mutant proteins. The amino acids encoded by the two target sequences are highlighted in red and purple. *, the stop codon. In **A** and **B**, the relative positions of nucleotides and amino acids are given next to the sequence. The sequence gap length (bp or aa) is shown in parentheses. (**C**) Flowers of the WT. (**D**) Flowers of *pfpos3*-L3. (**E**) Carpels of the WT. (**F**) Carpels of *pfpos3*-L3. (**G**) Seeds of the WT. (**H**) Seeds of *pfpos3*-L3. (**I**) Mature fruit of the WT. (**J**) Mature fruit of *pfpos3*-L3. Bars = 1 cm in **C**–**J**. (**K**) Fruit weight variation of the WT and the three *pfpos3* lines. (**L, M**) Epidermal cells of floral organs and mature berries of the WT (L) and *pfpos3*-L3 (M). 1–5 represent the sepal (adaxial surface at the basal region), petal (adaxial surface at tip area), stamen (middle region of abaxial surface), carpel (middle region of dorsal surface) from mature flowers, and mature berries, respectively. Bars = 100 μm. (**N**) Cell size variation of four whorl floral organs and mature berries. (**O**) Diagram of the transverse section of fruit. p1 and p2 indicate different parts of the pericarp for cell analysis. (**P**) Paraffin sections of WT and *pfpos3*-L3 berries 0 DAF around p1. (**Q**) Paraffin sections of WT and *pfpos3*-L3 berries 0 DAF around p2. Bars = 100 μm in **P** and **Q**. (**R**) Paraffin sections of WT and *pfpos3*-L3 berries 5 DAF around p1. (**S**) Paraffin sections of WT and *pfpos3*-L3 berries 5 DAF around p2. DAF, days after fertilization. Bars = 100 μm in **R** and **S**. (**T**) Cell size variation around p1 and p2 of berries 0 DAF and 5 DAF. In **K**, **N**, and **T**, the mean and S.D. are presented. Sample size was given below each column. ^*^, *P* < 0.05; ^**^, *P* < 0.01 in two-tailed Student’s *t*-test.

## Results

### *POS3-*like belonged to the LBD gene family

To reveal functional divergence of the *POS3-*like genes between Physalis and Solanum, the full-length sequences *PfPOS3*, *SpPOS3*, and *SlPOS3* were isolated from *P*. *floridana*, *S*. *pimpinellifolium*, and *S*. *lycopersicum*, respectively. They had an identical gene structure, featuring one intron and two exons (Fig. S1A). *SpPOS3* and *SlPOS3* were identical in the CDSs and genomic sequences, and the identities of *PfPOS3* with these Solanum orthologs in the CDSs, genomic sequences, and amino acids were 85.02%, 63.61%, and 81.45%, respectively. The putative PfPOS3, SpPOS3, and SlPOS3 proteins contained a conserved LOB domain (Fig. S1B) and SpPOS3, and SlPOS3 were LOB30 in Solanum (Fig. S1C). Searching the *P*. *floridana* genome [25], a total of 40 LBD genes were identified, and phylogenetic analysis classified PfPOS3 into Class IB, grouped with SpPOS3 and SlPOS3 from Solanum (Fig. S2), suggesting that these *POS3-*like genes were likely orthologous to *AtLBD18* in *Arabidopsis*. The syntenic analysis of *POS3-*like in *P*. *floridana*, *S*. *pimpinellifolium*, and *S*. *lycopersicum* further confirmed the orthology of *PfPOS3*, *SpPOS3*, and *SlPOS3* (Fig. 1A). The high conservation of sequences and structures might imply a conserved role for *POS3-*like.

### Heterometric expression of *POS3-*like implicated a role in flower and fruit development in *P. gloridana*

To elucidate the potential roles of *POS3-*like, we first investigated its mRNA expression in developing fruit at multiple developmental stages in *P*. *floridana*, *S*. *pimpinellifolium*, and *S*. *lycopersicum*. qRT-PCR analysis clearly showed that the expression level of *PfPOS3* was significantly higher than that of *SpPOS3* and *SlPOS3* in developing fruit, which was elevated as the fruit developed (Fig. 1B). We then conducted *in situ* hybridization to detect the cellular distribution of *POS3-*like mRNA in floral buds and fruits. During floral organ initiation and morphogenesis, *PfPOS3* was heavily expressed in the floral meristem, primordia, and early development of floral organs (Columns 1–5 in Fig. 1C). After fertilization, the expression signals of *PfPOS3* were detected in the developing fruit, especially in the seeds (Columns 6–7 in Fig. 1C). The observed expression in *P*. *floridana* suggests a role for *PfPOS3* in floral and fruit development. In contrast, *SpPOS3* expression was not detected during floral and fruit development (Columns 1–7 in Fig. 1D), and *SlPOS3* expression was detected only in the seeds of the developing fruit (Columns 1–7 in Fig. 1E). The *POS3-*like sense probes did not show any signal in the flower buds (Column 8 in Fig. 1C–E). The mRNA accumulation patterns of *SpPOS3* and *SlPOS3* were consistent with the results of the Tomato Expression Atlas (Fig. S3). However, these POS3-like proteins shared identical subcellular localization, and they were localized in the nucleus and cytoplasm (Fig. S4). The mechanism underlying such a heterometric expression of *POS3-*like mRNA in *P. floridana* relative to *S. pimpinellifolium* and *S*. *lycopersicum* might lie in the striking alterations in the predicted *cis*-regulatory motifs of their promoters and trans-acting factors from library screens (Fig. S5, Table S1), which needs further investigation, but the heterometry, characterized by a significantly elevated expression of *PfPOS3* compared to its orthologous genes within Solanum, implied a role of this gene in flower and fruit development of Physalis.

### *PfPOS3* positively regulated reproductive organ size in *P*. *floridana*

To reveal the developmental role of *PfPOS3*, gene editing via the CRISPR/Cas9 system was first conducted to target the first exon of *PfPOS3* (Fig. 2A). Three homozygous and independent gene-edited plant lines were analyzed in *P*. *floridana*. Compared with the WT, *pfpos3*-L1 and *pfpos3*-L2 had a 1-bp deletion, and *pfpos3*-L3 produced a 274-bp deletion between the two targets, and these indels led to premature stop codons and complete loss of the LOB domain (Fig. 2A and B), thus presumably being loss-of-function mutations. Relative to the WT, the flower radius was reduced by about 3% in *pfpos3* plants (Fig. 2C and D, Table S2), and carpels 0 DAF and seed weight of the *pfpos3* lines became small (Fig. 2E–H, Table S2). The mature fruit of the three *pfpos3* lines was significantly smaller than that of the WT, with the fruit weight reduced by 13%–26% (Fig. 2I–K). Therefore, *PfPOS3* may act as a promoter of reproductive organ size.

To further understand how organ size changed, we analyzed cell variations. The epidermal cell size of the examined floral organs from mature flowers in these *pfpos3* lines was significantly smaller than that of the WT, of which the epidermal cells of carpels 0 DAF decreased by about 7% (Fig. 2L–N, Table S2) and the epidermal cells of mature berries were also decreased by about 16% (Fig. 2L–N, Table S6). The developing berries 0 and 5 DAF were transverse-sectioned, and the cell size was evaluated in different mesocarp positions (Fig. 2O). The mesocarp cell size of these developing berries was significantly smaller than that of the WT berries at the corresponding developmental stages (Fig. 2P–T, Table S2). However, the decrease rate in cell size did not fully account for the size reduction in the pericarp area and fruit size since the cell number increased (Table S2). Therefore, *PfPOS3* regulated floral organ size and fruit weight by promoting cell size and inhibiting cell division.

The observed *PfPOS3* roles were further endorsed by generating *PfPOS3*-RNAi transgenic Physalis lines. Similar to *pfpos3* plants, we observed that the floral organ size and seed weight of *PfPOS3*-RNAi were smaller than those of the WT (Fig. S6A–F, Table S3). The mature fruit of the *PfPOS3*-RNAi lines was also smaller than that of the WT, with fruit weight reduced by 4%–22% (Fig. S6G–J, Table S3). The sizes of the epidermal cells of the examined floral organs and mature berries and the internal cells of developing fruit 0 and 5 DAF were significantly smaller than those of the WT, while the cell number was increased (Fig. S7, Tables S3 and S6).

### Overexpressing *PfPOS3* and *SlPOS3* generated similar flower and fruit variation

**Figure 3:**
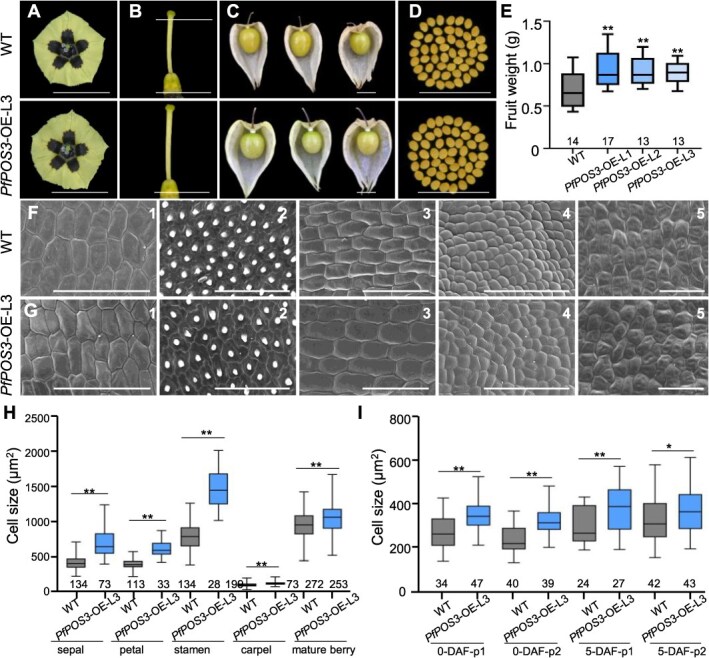
Phenotypic analysis of transgenic *PfPOS3*-OE Physalis plants. (**A**–**D**) Flowers, carpels, mature fruits, and seeds of the WT and *PfPOS3*-OE-L3. Bars = 1 cm. (**E**) Fruit weight variation of the WT and the three *PfPOS3*-OE lines. (**F, G**) Epidermal cells of floral organs and mature berries of the WT (F) and *PfPOS3*-OE-L3 (G). 1–5 represent the sepal (adaxial surface at the basal region), petal (adaxial surface at tip area), stamen (middle region of abaxial surface), carpel (middle region of dorsal surface) from mature flowers, and mature berries, respectively. Bars = 100 μm. (**H**) Cell size variation of four whorl floral organs and mature berries. (**I**) Cell size variation around p1 and p2 (defined in Fig. 2O) of berries 0 DAF and 5 DAF by paraffin sections. In **E**, **H**, and **I**, the mean and S.D. are presented. Sample size was given below each column. ^*^, *P* < 0.05; ^**^, *P* < 0.01 in two-tailed Student’s *t*-test.

To further confirm the function of *PfPOS3*, we overexpressed this gene in *P*. *floridana* using the *35S* promoter. Three independent *PfPOS3*-overexpressing (*PfPOS3*-OE) transgenic lines exhibiting similar variation in reproductive organs were selected for subsequent analyses (Fig. S8A). In the transgenic Physalis plants obtained, floral organ size and mature fruit/seed weight showed a significant increase compared with the WT (Fig. 3A–E, Table S4). The fruit weight was increased around 29%–35% in the three *PfPOS3*-OE lines (Table S4). The epidermal cell size of floral organs and mature berries and the size of internal cells of developing fruit 0 and 5 DAF were significantly larger than those of the WT (Fig. 3F–I, Tables S4 and S6), while the cell number was reduced (Tables S4 and S6). These observations confirmed that *PfPOS3* promoted cell size and inhibited cell division in reproductive organs.

To exclude potential influence of the coding sequence variation on the *POS3-*like function, we overexpressed *SlPOS3* CDS in *P*. *floridana*, and three independent *SlPOS3*-OE transgenic *P*. *floridana* plant lines exhibiting similar variation in reproductive organs were analyzed (Fig. S8B). Phenotypic variations in floral organs, seeds, and mature fruit in these *SlPOS3*-OE transgenic *P*. *floridana* plant lines were similar to *PfPOS3*-OE (Fig. S8C–G, Table S5). The size of the epidermal cells of the examined floral organs from mature flowers and mature berries was significantly larger than those of the WT (Fig. S8H–K, Tables S5 and S6). The size of internal cells of developing fruits was also significantly larger than that of the WT in *SlPOS3*-OE, and cell division was inhibited (Table S5), as observed in *PfPOS3*-OE. These observations suggested that *SlPOS3* had similar roles as *PfPOS3* did in transgenic Physalis plants.

To endorse this presumption, we investigated cell variation during fruit development in all transgenic *POS3-*like Physalis plants. In *pfpos3* and *PfPOS3*-RNAi, the cells were significantly smaller while the number of cells increased. Conversely, the cells were significantly larger and the number of cells decreased in all obtained *POS3-*like overexpressed plants (Table S6). These observations further supported the role of these *POS3*-like in cell division and cell expansion.

### POS3-like interacted with TCP15 and TCP18

LBD proteins usually perform biochemical and biological functions by forming dimers or complexes [4]. To identify the interacting proteins of POS3-like, Y2H analyses were first performed. No toxicity was observed for POS3-like proteins as baits in yeast, but they showed different extents of auto-activation, particularly having a strong auto-activation activity for PfPOS3 (Fig. S9A). The *S*. *pimpinellifolium* and *S*. *lycopersicum* expression libraries were screened directly using Solanum POS3-like since weak autoactivation was completely repressed by adding 10 mM/L 3-AT, while truncated PfPOS3 (only LOB domain) was used as bait in the *P*. *floridana* expression library screens (Fig. S9B and C). On these library screens, we found two teosinte branched1/cycloidea/proliferating cell factors (TCPs) designated TCP15 and TCP18 TFs as the best candidate interacting proteins of POS3-like.

**Figure 4:**
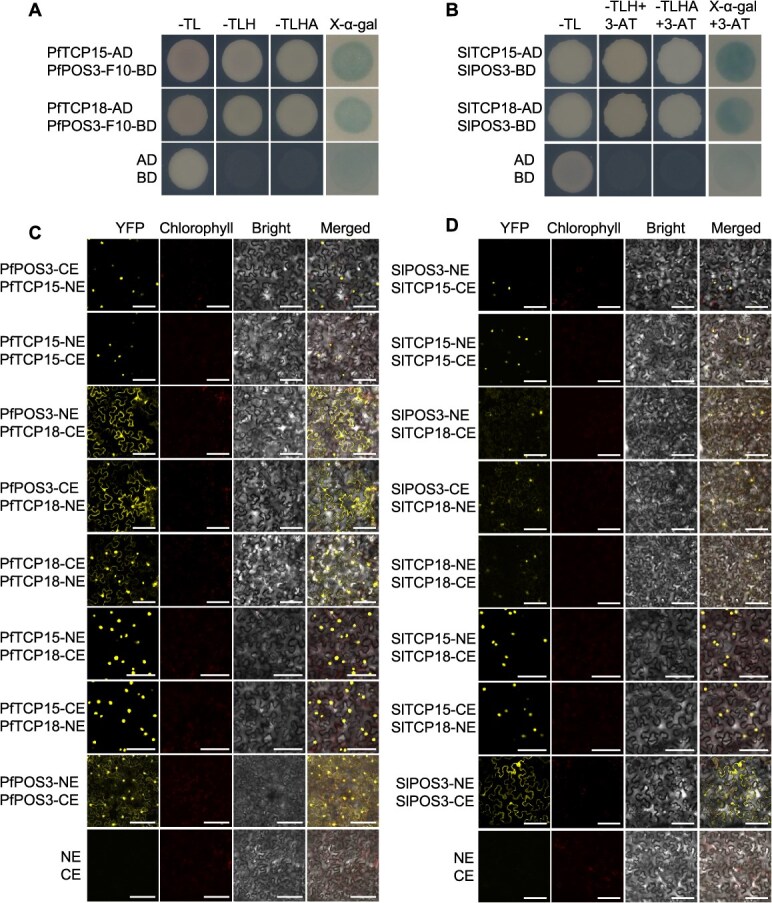
Dimerization among POS3-like, TCP15, and TCP18. (**A**) Interactions of PfPOS3 and PfTCPs in yeast. Partial PfPOS3 (F10) was used because of the self-activation of PfPOS3 (Fig. S9). (**B**) Interactions of SlPOS3 and SlTCPs in yeast. -TL, synthetically defined medium (SD)/-Trp-Leu; -TLH, SD/−Trp-Leu-His; -TLHA, SD/-Trp-Leu-His-Ade; X-α-gal, SD/-Trp-Leu-His-Ade + X-α-gal. Ten millimolar 3-AT was added in the detection of the PPIs between SlPOS3 and SlTCPs due to the weak auto-activation of SlPOS3. (**C**) PPIs of PfPOS3 and PfTCPs revealed by BiFC in tobacco leaves. (**D**) PPIs of SlPOS3 and SlTCPs revealed by BiFC in tobacco leaves. NE, N-terminal fragment of yellow fluorescent protein (YFP); CE, C-terminal fragment of YFP. Bars = 100 μm.

To confirm the detected protein–protein interactions (PPIs), we isolated the CDSs of *TCP15* and *TCP18* from *P*. *floridana*, *S*. *pimpinellifolium*, and *S*. *lycopersicum*. Their putative proteins had a typical TCP domain shared with the closest homologs, AtTCP7 and AtTCP20 from *Arabidopsis* (Fig. S10A and B). TCP15 and TCP18 from Physalis showed sequence divergence around 70%, compared to their Solanum orthologs, and each orthologous gene pair (*SpTCP15*/*SlTCP15* and *SpTCP18*/*SlTCP18)* from the two Solanum species was identical in CDS (Fig. S10C and D). The interactions of POS3-like and these TCPs were verified in yeast cells (Fig. 4A and B). They were further investigated in *N*. *benthamiana* leaf epidermal cells via BiFC assays. Based on the YFP signals (the interaction) observed, we found that the three Physalis TFs (POS3 and two TCPs) formed homodimers, and they all formed heterodimers (Fig. 4C and D). Moreover, YFP signals were observed in the nucleus in the PfTCP15-related interactions, and in both the nucleus and cytoplasm in the interaction of PfPOS3 and PfTCP18 (Fig. 4C). Similar interacting patterns were observed for Solanum POS3-like and TCPs (Fig. 4D). These results suggest conserved interactions between POS3-like and the two identified TCPs from *P. floridana*, *S. pimpinellifolium*, and *S*. *lycopersicum*.

### Co-expression of *TCP15*, *TCP18*, and *POS3-*like

**Figure 5:**
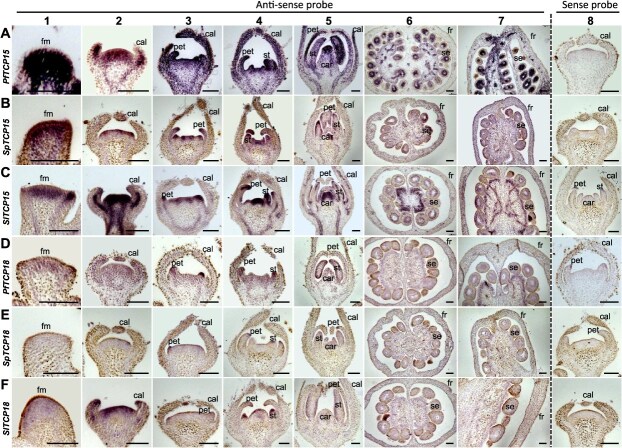
*In situ* hybridization of *TCP15* and *TCP18* in three solanaceous species. (**A**) *PfTCP15*. (**B**) *SpTCP15*. (**C**) *SlTCP15*. (**D**) *PfTCP18*. (**E**) *SpTCP18*. (**F**) *SlTCP18*. Floral organs and berries from *P*. *floridana*, *S*. *pimpinellifolium*, and *S*. *lycopersicum* 0 DAF were hybridized by antisense (1–7) and sense (8) probes for each gene, as indicated. 1–7 represents the stages of floral primordia, sepal primordia, petal primordia, stamen primordia, carpel primordia, fruit 0 DAF (transverse cutting), and fruit 0 DAF (longitudinal cutting), respectively. Eight, respectively, represents the various primordia stages. fm, floral meristem; cal, calyx primordia; pet, petal primordia; st, stamen primordia; car, carpel primordia; fr, fruit; se, seed. Bars = 100 μm.

To further understand the biological pertinence of the detected PPIs, we detected the expression of these *TCP* genes in the three species. qRT-PCR results demonstrated expression patterns during fruit development (Fig. S11A and B). *TCP15* was basically expressed in all stages examined in *P. floridana*, *S. pimpinellifolium*, and *S*. *lycopersicum* (Fig. S11A), while the *TCP18* orthologous genes had an obvious elevated expression during fruit development (Fig. S11B). In particular, relative to the Solanum orthologous genes, *PfTCP18* was highly expressed and upregulated as the fruit developed (Fig. S11B). Using *in situ* hybridization, the expression of *PfTCP15*, *SpTCP15*, and *SlTCP15* was detected in the floral meristem, floral organ primordium, and developing fruit/seeds (Columns 1–7 in Fig. 5A–C). All orthologous *TCP18* genes were also detected in these domains (Columns 1–7 Fig. 5D–F). *TCP15* and *TCP18* sense probes did not produce any signals in the flowers (Column 8 in Fig. 5). All results were consistent with the Tomato Expression Atlas (Fig. S11C–F) and previous work [31]. We further investigated the subcellular localization of these TCPs and found that TCP15 proteins from *P. floridana*, *S. pimpinellifolium*, and *S*. *lycopersicum* were mainly localized in the nucleus, while TCP18 proteins were localized in both the nucleus and cytoplasm (Fig. S12). These results suggested the co-expression of the TCPs and POS3-like in mRNA accumulation and subcellular localization, which strongly supported the functional relevance of physical interactions between TCP15, TCP18, and POS3-like in fruit development.

### POS3-TCP modules regulated the expression of *CYCD1;1* and *CYCB1;1*

Considering heterodimerizations among POS3-like, TCP15, and TCP18 and their roles in fruit development, they may share common downstream target genes in cell cycle control. *CYCD1;1* and *CYCB1;1* are the direct target genes of *Arabidopsis* TCP7 and TCP20 [32, 33], respectively, and the orthologs of Solanum TCP15 and TCP18 [31], respectively, which are further supported by our phylogenetic analysis (Fig. S13). In *POS3-*like-related transgenic plants, the expression of *TCP15*, *TCP18*, *CYCD1;1*, and *CYCB1;1* was indeed significantly changed (Fig. S14A). First, the expression levels of *TCP15* and *TCP18* were significantly increased in *POS3-*like overexpressed plants; while significantly decreased in *pfpos3* and *PfPOS3*-RNAi, indicating a positive regulation of these TCP genes by POS3-like. Second, *CYCD1;1* expression increased in all *POS3-*like-overexpressing plants and significantly decreased in *PfPOS3* knockdown and knockout plants. Third, *CYCB1;1* expression significantly decreased in all *POS3-*like overexpressing plants and was activated in *PfPOS3* knockdown and knockout plants. Particularly, these cyclin genes, as downstream cell control pathways, are the primary putative direct target genes of POS3-like, TCP15, and TCP18.

**Figure 6:**
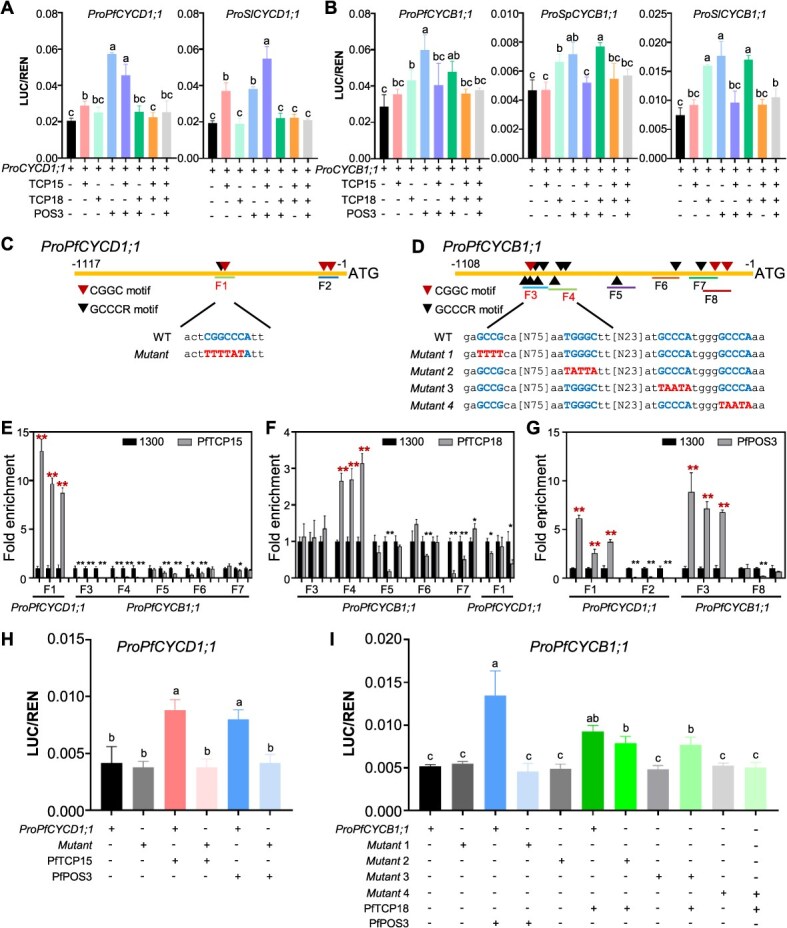
POS3-TCPs regulate the expression of *CYCD1;1* and *CYCB1;1*. (**A**) Transcriptional activity of POS3-TCPs on the promoters of *CYCD1;1* in *P*. *floridana* petal protoplasts. (**B**) Transcriptional activity of POS3-TCPs on the promoters of *CYCB1;1* in *P*. *floridana* petal protoplasts. Information on the effects and reporters is presented in Fig. S14. (**C**) Schematic structure of *ProPfCYCD1;1*. (**D**) Schematic structure of *ProPfCYCB1;1*. In **C** and **D**, red triangle, location of CGGC motif; black triangle, location of GCCCR motif. F1–F8, fragments detected by ChIP-qPCR. WT, promoter sequences of *PfCYCD1;1* or *PfCYCB1;1*; *Mutant*, and *Mutant1–4*, sequence of site-directed mutagenesis. (**E**) ChIP-qPCR analysis of PfTCP15 binding to fragments containing the GCCCR motif. (**F**) ChIP-qPCR analysis of PfTCP18 binding to fragments containing the GCCCR motif. (**G**) ChIP-qPCR analysis of PfPOS3 binding to fragments containing the CGGC motif. In **E**–**G**, ChIP-PCR for each fragment was performed using three independent samples. The mean and S.D. are presented. *, *P* < 0.05; **, *P* < 0.01 in two-tailed Student’s *t*-test. Only the red stars indicate the enriched fragments. (**H**) Transcriptional activity of PfPOS3 and PfTCP15 on mutated *ProPfCYCD1;1* in *P*. *floridana* petal protoplasts. (**I**) Transcriptional activity of PfPOS3 and PfTCP18 on mutated *ProPfCYCB1;1* in *P*. *floridana* petal protoplasts. In **A**, **B**, **H**, and **I**, the mean and S.D. are presented (*n* = 3). The same lowercase letter indicates no significant difference, while different lowercase letters represent a significant difference at *P* < 0.05 in the two-tailed Student’s *t*-test. LUC, firefly luciferase; REN, *Renilla* luciferase.

To substantiate this, approximately 1.0-kb putative promoters of *CYCD1;1* and *CYCB1;1* were tested to be active since the GFP gene driven by them was expressed (Fig. S14B–G), and they were further used in transient *LUC* expression analyses. In the LUC tests, the three TFs from *S. pimpinellifolium*, *S*. *lycopersicum*, and *P. floridana* were used to make effector constructs, while the promoters of the involved *CYC* genes were set as reporters (Fig. S14H). All effectors and reporters were functional in tobacco leaf cells (Fig. S14H and I). For precise quantification, we ran the transient expression in Physalis protoplasts from petals and found that effectors associated with POS3-like, TCP15, and their heterodimers positively regulated *LUC* expression when the *CYCD1;1* promoter was used as a reporter, while other combinations of these TFs did not have such an effect (Fig. 6A). In particular, TCP18 repressed the activating effects of POS3-like and TCP15 on *CYCD1;1* expression (Fig. 6A). When the *CYCB1;1* promoter was used as a reporter, TCP18 and POS3-like were major effectors upregulating *LUC* expression. Other combinations of these TFs did not have such an effect, but TCP15 repressed the activating effects of POS3-like and TCP18 on *CYCB1;1* expression (Fig. 6B). Moreover, the regulation of downstream *CYC* genes by POS3-like, TCPs, and their heterodimers was conserved (Fig. 6A and B).

*Arabidopsis* TCP7 and TCP20 bound to the GCCCR motif in the *CYCD1;1* and *CYCB1;1* promoters, respectively, to regulate their expression [32, 33], and LBD TFs bound to the (G)CGGC(G) motif [34]. Therefore, we analyzed these motifs in the *CYCD1;1* and *CYCB1;1* promoters in Physalis. One GCCCR motif and three (G)CGGC(G) motifs were found in *ProPfCYCD1;1* (Fig. 6C), and 11 GCCCR motifs and three (G)CGGC(G) motifs were found in *ProPfCYCB1;1* (Fig. 6D). To determine the binding sites of PfPOS3, PfTCP15, and PfTCP18 in the putative promoter of *PfCYCD1;1* or *PfCYCB1;1*, ChIP-qPCR aimed at these putative motifs was performed. For this purpose, eight fragments designated F1–F8 were designed based on the distribution of these putative binding motifs (Fig. 6C and D). Only F1 of *ProPfCYCD1;1* was highly enriched when *PfTCP15* was overexpressed (Fig. 6E), while only F4 of *ProPfCYCB1;1* was highly enriched in the *PfTCP18*-overexpressing leaves (Fig. 6F). F1 of *ProPfCYCD1;1* and F3 of *ProPfCYCB1;1* were highly enriched in the *PfPOS3*-overexpressing leaves (Fig. 6G). These results revealed that the *cis*-regulatory motifs on F1, F3, and F4 may form the basis for directly regulating *cyclin* genes by these TFs.

To further clarify the specific role of these predicted *cis*-motifs, site-directed mutagenesis was performed using *P*. *floridana* genes. Several versions (*Mutant* and *Mutant1–4*) of *ProPfCYCD1;1* and *ProPfCYCB1;1* targeting the predicted motifs on the identified functional fragments (F1, F3, and F4) were generated (Fig. 6C and D). One LBD-binding motif and one TCP-binding motif were predicted on F1, and the two *cis*-motifs overlapped, and the designed nucleotide variation (*Mutant*) simultaneously disrupted the two motifs (Fig. 6C). The regulatory effect of PfPOS3 and PfTCP15 was abolished once the predicted motifs in F1 were mutated (Fig. 6H), suggesting that the two TFs indeed bound directly to the motifs of the *PfCYCD1;1* promoter. One LBD-binding motif and three TCP-binding motifs were predicted in F3 and F4 of the *PfCYCB1;1* promoter, and they were mutated to be *Mutant1–4* (Fig. 6D). Site-directed mutagenesis revealed that PfPOS3 bound to the first motif (*Mutant1*) on F3, while PfTCP18 bound to the third mutated motif (*Mutant4*) on F4 of the *PfCYCB1;1* promoter (Fig. 6I).

Taken together, these results suggest that PfPOS3, TCPs, and PfPOS3-TCP complexes directly regulate the expression of *CYCD1;1* and *CYCB1;1* via specific selection of the *cis*-elements, thus regulating cell size and cell division.

### Genetic interactions of *PfPOS3* and *PfTCP*s in fruit development

**Figure 7:**
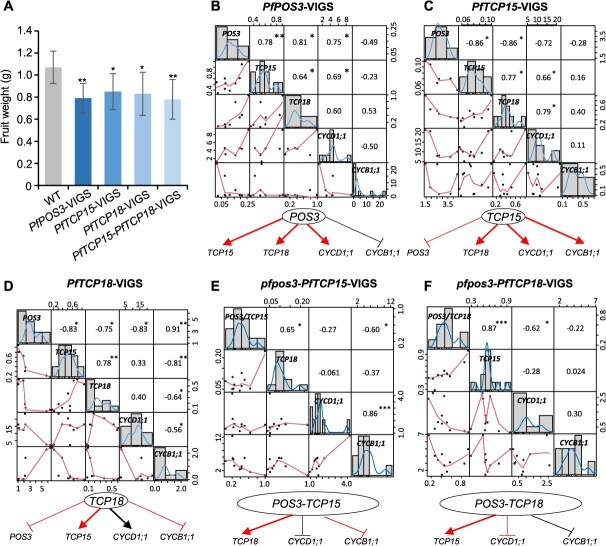
Virus-induced gene silencing (VIGS) analyses of *PfPOS3* and *PfTCPs* in *P*. *floridana*. (**A**) Fruit weight variation of the VIGS treatments (*POS3*, *TCP15*, *TCP18*) in the WT *P. floridana*. *, *P* < 0.05; **, *P* < 0.01 in two-tailed Student’s *t*-test. (**B**–**D**) Correlation matrix of gene expression (*POS3*, *TCP15*, *TCP18*, *CYCD1;1*, and *CYCB1;1*) in the WT *P. floridana*. (**B**) *PfPOS3*-VIGS. (**C**) *PfTCP15*-VIGS. (**D**) *PfTCP18*-VIGS. (**E, F**). Correlation matrix of gene expression (*POS3*, *TCP15*, *TCP18*, *CYCD1;1*, and *CYCB1;1*) in the *pfpos3*. (**E**) *pfpos3*-*PfTCP15*-VIGS. (**F**) *pfpos3*-*PfTCP18*-VIGS. In B–F, the distribution of each variable is shown on the diagonal, and the bivariate scatter plots with a fitted line are displayed. The deduced gene regulations are given below each matrix. The arrow indicates a positive correlation, and the T line indicates a negative correlation. The red indicates a *P*-value <0.05; the black indicates *P*-value >0.05. The Pearson correlation coefficient was calculated in SPSS, and the correlation matrix was visualized using PerformanceAnalytics (https://CRAN.R-project.org/package=PerformanceAnalytics). ^*^, *P* < 0.05; ^**^, *P* < 0.01; ^***^, *P* < 0.001 in two-tailed Student’s *t*-test.

To reveal the biological relevance of the PPIs and regulations associated with the three TFs in fruit development, we constructed a series of VIGS lines of these genes in the *P*. *floridana* (WT) and *pfpos3* backgrounds. The variation in gene expression and related traits was evaluated in these VIGS treatments (Table S7), and their relationships were further evaluated. In the WT background, once *PfPOS3* was first knocked down via VIGS, the flower size, fruit weight, and seed weight decreased (Fig. 7A, Table S7). Moreover, in our statistical analysis, the variation in gene expression and the deduced regulatory pattern in VIGS experiments showed that *PfPOS3* might activate *PfTCP15*, *PfTCP18*, and *PfCYCD1;1* and repress *PfCYCB1;1* (Fig. 7B). The variation in morphology and the deduced regulatory pattern was similar to that observed in *PfPOS3*-RNAi and *pfpos3*; therefore, the developmental role of *TCPs*, and their interactions with *PfPOS3* were further exploited in *P*. *floridana* using the VIGS approach. In *PfTCP15* and *PfTCP18* knockdowns, the fruit weight was significantly reduced relative to the WT (Fig. 7A, Table S7), and the deduced regulations were that *PfTCP15* repressed *PfPOS3* but activated other related genes (Fig. 7C) and *PfTCP18* repressed *PfPOS3* and *PfCYCB1;1* and activated *PfTCP15* and *PfCYCD1;1* (Fig. 7D). We further silenced the two *TCP* genes and observed that *PfPOS3* and the two *CYC* genes were repressed in WT *P. floridana* (Fig. S15A) and that the fruit weight had a tendency to be small (Fig. 7A, Table S7). These results suggest that the two *TCP* genes were directly or indirectly activated by *PfPOS3*, which in turn directly or indirectly repressed *PfPOS3* in fruit development. Thus, we assumed a developmental role of POS3-TCP complexes or their genetic interactions in fruit size control in *P. floridana*.

To verify these assumptions, we knocked down the two *TCPs* in *pfpos3* backgrounds. However, relative to *pfpos3*, further downregulation of one or two *TCP* genes did not lead to a further deviation in fruit weight from that of *pfpos3* (Fig. S15B), implying that the observed TCPs’ regulations on fruit weight may require PfPOS3, namely forming POS3-TCP complexes. We further evaluated the potential regulation of the heterodimers of three TFs on *CYC* genes in these VIGS treatments and found that the POS3-TCP15 and POS3-TCP18 complexes repressed *CYCD1;1* and *CYCB1;1* expression (Fig. 7E and F). Notably, the regulations on *CYCD1;1* and *CYCB1;1* expression in *pfpos3*-TCP15-TCP18-VIGS resembled that in *pfpos3* and *PfPOS3*-VIGS (Figs. 7B, S14A, and  S15C). These results further supported the revealed regulations of these TFs on *CYC* genes via putative POS3-TCP complexes. Therefore, *PfPOS3* and *TCP* genes interacted genetically and regulated fruit development by recruiting POS3-TCP modules in *P. floridana*.

## Discussion

The developmental role of LBD has been revealed in multiple developmental processes in plants, and only a few cases have been reported in fruit development. In this work, we functionally characterized LBD gene *PfPOS3* in *P. floridana* and found that it regulated flower size and fruit weight by promoting cell expansion and inhibiting cell division. Comparative analyses revealed that *PfPOS3* and its orthologous genes from *S. pimpinellifolium* and *S*. *lycopersicum* were conserved in sequences and molecular interactions with two TCP-CYC pathways; however, distinct heterometric (strongly elevated) expression of *PfPOS3* in *P. floridana* relative to its orthologs in *S. pimpinellifolium* and *S*. *lycopersicum* was observed, which may suggest that SlPOS3 has diverged from a role in governing flower and fruit development within Solanum.

### *PfPOS3* regulates reproductive organ size

**Figure 8:**
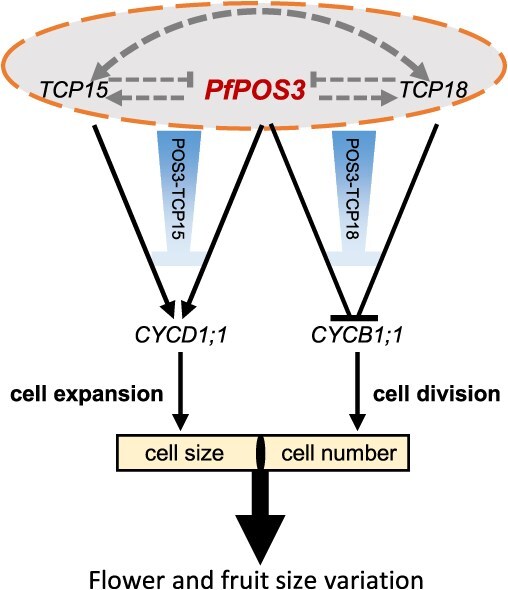
Working model of *PfPOS3* in developmental evolution of fruit weight and flower size. The proposed working model for *PfPOS3* recruiting POS3-TCP-CYC modules regulating fruit weight variation. PfPOS3 interacted with TCPs to form POS3-TCP modules to fulfil the related regulations. The arrow represents activation, while the T lines indicate repression. The dashed line or dashed orange circled gray area indicates regulations needing verification.

The LBD gene family is a large gene family with more than 30 members in various plant species [2, 35–37]. They play important roles, as summarized in the section Introduction; however, only four studies revealed the role of LBDs in regulating banana fruit ripening [38], tomato fruit softening [12], and tomato fruit development [13, 14]. We previously identified *P5* as a DEG upon fertilization between *S. pimpinellifolium* and *P. floridana* through transcriptomic comparisons and showed a potentially negative correlation between the expression of this gene and mature fruit size in VIGS [30]. In accordance with these findings, the present study comprehensively demonstrated that *PfPOS3*, encoding an LBD protein, was heavily expressed in floral meristems, floral organs, and developing fruit in *P. floridana*. This was revealed through a combination of mRNA *in situ* hybridizations and detailed qRT-PCR assays. To further reveal its developmental roles, we generated *PfPOS3*-Crispr, *PfPOS3*-RNAi, and *PfPOS3*-OE transgenic Physalis plants. Genotypic variations in these transgenic plant lines revealed that floral organ size and fruit weight decreased significantly in loss-of-function mutations of *PfPOS3* (*pfpos3)* and *PfPOS3*-RNAi transgenic Physalis plants, suggesting an essential role of *PfPOS3* in flower and fruit development. In corroboation with this, *PfPOS3* overexpression significantly promoted floral organ and fruit size. *PfPOS3* mainly promoted cell size, and intrinsically orchestrate cell expansion and cell division during fruit development (Fig. 8). However, these findings reject the presumption that *P5* could be a negative regulator of reproductive organ size in VIGS [30]. This may be due to the limitation of gene expression data from semi-flowers, which did not correspond precisely to mature fruits that we measured because of the characteristics inherent to VIGS; for example, it often leads to chimeric organs [39]. While VIGS may serve as an efficient method for revealing the developmental pathways of a gene of interest [39], the precise developmental role of the investigated gene requires verification through additional sophisticated methodologies, such as gene editing. In this work, we convincingly demonstrated the positive role of *PfPOS3* in floral organ and fruit size control.

LBD and TCP genes are involved in the control of flowering time [40–43] and the specification of floral organ identity in various plant species [44–47]. However, in the transgenic Physalis lines related to *PfPOS3* that were obtained, neither flowering time nor floral organ identity was altered when compared to WT plants. Additionally, these genes have been reported to affect the fruit quality, including variation in berry softening [12, 48, 49] and anthocyanin accumulation [50, 51]. Nevertheless, the roles of *POS3* and TCPs in fruit quality in terms of nutritional content and secondary metabolites merit to be investigated in Physalis.

### *PfPOS3* is integrated into the TCP-CYC pathways

As a TF, LBD can interact with other TFs or directly bind to the promoter of downstream genes to regulate their expression. The TCP-CYC pathways are well characterized to regulate the cell cycle [52]. CINCINNATA (CIN)-TCPs interact with LBD protein ASYMMETRIC LEAVES 2 (AS2) to regulate cell proliferation during leaf development by suppressing the expression of the *KNOX* gene in *Arabidopsis* [53]. In our study, we found PPIs between PfPOS3 and two TCP proteins (TCP15 and TCP18). TCP genes are also plant-specific TFs involved in diverse growth-related processes, such as embryonic growth, leaf development, floral organ morphogenesis, and cell cycle regulation [52, 54]. AtTCP7 and AtTCP20, as the corresponding orthologues of Solanum TCP15 and TCP18 [31], are essential components of the TCP-CYC pathways [32, 33]. Therefore, *PfPOS3* is apparently integrated into the TCP-CYC pathways in fruit development by assembling the POS3-TCP (LBD-TCP) modules. We found that homodimerization and heterodimerization occurred among PfPOS3, TCP15, and TCP18 and that reciprocal regulations between *PfPOS3* and the two TCP genes may exist, which could make the molecular situation complicated. Little is particularly known about their reciprocal regulations. However, the effects on fruit weight variation after further downregulation of these *TCP* genes in WT and *pfpos3* implied the dominant effect of *PfPOS3* on regulating fruit weight and the role of heterodimers between PfPOS3 and TCPs (TCP15 and TCP18). Nonetheless, TCPs may exert a braking role on POS3 effects. This was supported by the observation that the expression of *PfPOS3* was significantly upregulated when *TCP15* and *TCP18* were knocked down. Moreover, such a braking role could be achieved by forming POS3-TCP modules to regulate *CYC* genes.

Cell cycle progression is driven by cyclin-dependent kinases (CDKs) that cooperate with *CYCs*. D-type *CYCs* are considered regulators of the G1-to-S transition, while B-type *CYCs* control the G2-to-M transition [55], thus potentially contributing to cell size and cell number. In *Arabidopsis*, AtTCP7 can directly bind to the promoter of *CYCD1;1* and induce *CYC* expression [33]. *AtTCP20* is a common regulator of many effectors involved in cell division and growth coordination that bind to cognate GCCCR elements in the *CYCB1;1* promoter [32]. These *CYC* genes were significantly altered in the related mutants of *PfPOS3* and *TCP* genes in *P*. *floridana*. The inferred regulatory patterns in *pfpos3* and *PfPOS3*-VIGS closely resembled those in *PfPOS3*-OE and *SlPOS3*-OE transgenic Physalis plants. This suggests a potential regulation of *CYCD1;1* and *CYCB1;1* by these TFs. We then tested the capability of the gene regulation of these TFs on *CYC* genes. First, POS3-like directly bound to the promoters of downstream genes *CYCD1;1* and *CYCB1;1* to activate and repress their expression. Second, TCP15 and TCP18 promoted and repressed the expression of *CYCD1;1* and *CYCB1;1*, respectively, by binding to their promoters. Third, these TFs could fulfill their regulation by binding to specific *cis*-elements, as revealed by our site-directed analyses. The mechanisms by which binding specificity is determined and precise regulation is achieved remain unclear; however, these TFs bind to responsive *cis*-elements to regulate the expression of these *CYC* genes at the transcriptional level.

Based on these observations, we concluded that the POS3-TCP15 and POS3-TCP18 complexes downregulated *CYCD1;1* and *CYCB1;1* expression (Fig. 8), which was different from the regulation of *CYC* genes by each of three TFs. Moreover, the regulations on *CYCD1;1* and *CYCB1;1* expression in *pfpos3*-TCP15-TCP18-VIGS resembled that in *pfpos3* and *PfPOS3*-VIGS. Therefore, the formation of putative heterodimers, even higher order complexes of among three TFs may guarantee precise regulation of the cell cycle during fruit development. Although in LUC analyses, the expression of *CYCB1;1* was promoted by POS3-like and POS3-TCP18, *CYCB1;1* expression was inhibited in *POS3-*like-OE transgenic plants and some VIGS treatments. The main reason for the observed regulatory difference *in vitro* and *in vivo* could be that the putative promoter of *CYCB1;1* used in our LUC analyses is insufficiently long enough to function as the native functional promoter of *CYCB1;1* in plants. Through establishing the two distinct POS3-TCP-CYC pathways (Fig. 8), *PfPOS3* regulated fruit weight by balancing both cell division and cell expansion.

Plant hormones play essential roles in cell division and cell expansion [56]. Notably, the crosstalk between the LBD-TCP module and plant hormone signaling pathways is known to influence organ development [57–59]. This implies an additional regulatory mechanism for the POS3-TCP modules in the fruit development of Physalis; however, this requires further investigation. We have proposed a working model for *PfPOS3* regulation of fruit size based on the evidence that we have gathered (Fig. 8). In the related regulatory pathways, PfPOS3 dominantly regulates the downstream genes, and the two TCP factors may function as brake regulators for *PfPOS3* mRNA expression or function in cell cycles. The heterodimers between the two TCPs and PfPOS3 mainly regulated the expression of *PfPOS3*, and further orchestrated cell size and cell number by balancing the expression of downstream gene *CYC*s. High *PfPOS3* expression promoted *TCP*s’ expression but repressed cell division (Fig. 8). Activated *TCP*s inhibited *POS3-*like, and genes involved in cell number and cell size control were strongly inhibited or promoted by forming different POS3-TCP modules. The developmental roles of these two TCP genes and the fine orchestration of the cell division and cell expansion by the POS3-TCP modules during fruit development apparently needs clarification via generating gene editing mutants, but they are no doubt involved in the size control of flower and fruit in Physalis.

### Implications of *POS3-*like in the developmental evolution of fruit size

The key to evolution is genetic change, which can have four basic consequences: heterochrony, heterotopy, heterometry, and heterotypy. Of these, ‘heterometry’ in evolution is relatively less reported. During bird evolution, the beak emerges as the dominant avian facial feature, adapting birds to diverse eco-morphological opportunities [60]. The expression of *BMP4*, the major driving force building beak mass, is higher in ducks than in chickens [61]. Fruit weight is not only a domestication trait for crops, but a trait determining seed dispersal in plants [17, 62]. *POS1* orthologous genes are co-opted for fruit weight control in Physalis and Capsicum within Solanaceae [27], and *POS1* expression levels are positively associated with weight variation in *P*. *philadelphica* berries [26]. In this study, heterometric (strongly elevated) expression of *POS3-*like was observed during fruit development in *P. floridana* relative to *S. pimpinellifolium* and *S. lycopersicum*. Moreover, *POS3-*like mRNA expression was elevated during fruit development. *POS3-*like consequently promoted cell size, floral organ size, and fruit weight and size via orchestrating cell size and cell number through establishing the POS3-TCP modules. *TCP*s’ braking effect on *PfPOS3* roles and the co-expression and coevolution of *PfPOS3* and the two *TCPs* in terms of gene expression may be very important for this orchestration. The mechanisms underlying the heterometric expression of *POS3-*like needs further investigation. However, ectopic expression of *POS3-*like may contribute to the evolution and development of fruit weight to a suitably size for dispersal in Physalis.

SlPOS3 from Solanum and PfPOS3 from Physalis shared high identity in sequences, subcellular localizations, and interactions with TCPs, and overexpressing *SlPOS3* gene in transgenic *P. floridana* phenocopied *PfPOS3*-OE plants, likely pinpointing the role of *POS3-*like in solanaceous flower size control and fruit weight variation. However, a substantial decrease in the expression of *SlPOS3* may lead to a pronounced weakening or complete loss of this function within Solanum, which needs investigations.

In conclusion, the LBD TF *POS3* is a positive regulator of reproductive organ size through promoting cell size and inhibiting cell division in *P. floridana*. PfPOS3 interacts physically with TCP15 or TCP18 to bind the promoter of *CYCD1;1* or *CYCB1;1* and to regulate the expression of these *CYC* genes, thus revealing the first case to recruit LBD-TCP modules for fruit weight control. We propose that the POS3-TCP-CYC regulatory pathways regulate the cell cycle in fruit development. *PfPOS3* expression in *P. floridana* is distinctly heterometric (strong) relative to its orthologous genes in *S. pimpinellifolium* and *S. lycopersicum*. The genetic variation and regulating mechanisms underlying this heterometry of *POS3-*like expression need investigation; nevertheless, such heterometry of elevated expression may contribute to determining flower size and fruit weight variation in Physalis. Our work sheds light on fruit evolution and provides valuable directions for crop breeding.

## Materials and methods

### Plant materials and growth conditions

*P. floridana* (P106), *S. pimpinellifolium* (LA1589), and *S. lycopersicum* (Heinz 1706) were grown in a greenhouse (IBCAS, Beijing, China). They were cultivated under long-day conditions (temperature 25–28°C, 16-hour light/8-hour dark). Plant materials were harvested, immediately frozen in liquid nitrogen, and stored at −80°C.

### qRT-PCR analysis

Total RNA was extracted from the tissues using the SV total RNA isolation system (Promega, Wisconsin, USA) and used for the first strand of complementary DNA (cDNA) synthesis with the PrimeScript™ RT reagent kit with gDNA Eraser (Takara, Dalian, China). Quantitative reverse transcription (qRT)-PCR analysis was performed using the SYBR® *Premix Ex Taq*™ kit (Takara, Dalian, China) using gene-specific primers (Table S8). Relative expression levels of the investigated genes were normalized compared to the expression levels of *PfActin* (pf04G108110), *SpActin* (Sopim03g078400), or *SlActin* (Solyc03g078400) using the 2^−ΔΔ*C*T^ method [63].

### Sequence isolation

Genomic sequences of *PfPOS3*, *SpPOS3*, and *SlPOS3*, and the putative *CYCD1;1* and *CYCB1;1* promoters were amplified from the genomic DNA of *P*. *floridana*, *S*. *pimpinellifolium*, and *S*. *lycopersicum*. The coding sequences (CDSs) of *POS3-*like, *TCP15*, and *TCP18* were isolated through reverse transcription (RT)-PCR using cDNA as templates. All involved PCRs were performed using gene-specific primers (Table S8). All isolated sequences were cloned into the *pEASY*®-Blunt Cloning Vector (TransGen Biotech, Beijing, China) for sequencing.

### Phylogenetic and syntenic analysis

Multiple amino acid sequence alignments were performed using Muscle in MEGA X [64]. Poorly aligned sequences were eliminated using G-blocks (http://molevol.cmima.csic.es/castresana/Gblocks_server.html). The maximum likelihood phylogenetic trees were constructed using IQ-TREE [65] with the Jones–Taylor–Thornton (JTT) model and 1000 replicates of bootstrap analysis. The obtained phylogenetic trees of the gene families (Table S9) were visualized using FigTree (http://tree.bio.ed.ac.uk/software/figtree/). Synteny was constructed and visualized using MCscan (Python version) [66].

### mRNA *in situ* hybridization

Various floral buds were fixed with paraformaldehyde (Sigma, St. Louis, USA), dehydrated in different concentration gradients of alcohol, and embedded in Paraplast Plus (Sigma, St. Louis, USA) according to a previous description [67]. Tissue sections (10 μm thick) were mounted on ProbeOn Plus Microscope Slides (Fisher Scientific, Massachusetts, USA). To synthesize probes labeled with digoxigenin according to the DIG-RNA Labeling Kit (SP6/T7) manual (Roche, Mannheim, Germany), 285-bp *PfPOS3*-, 310-bp *SlPOS3*-, 365-bp *PfTCP15*-, 387-bp *SlTCP15*-, 490-bp *PfTCP18*-, and 456-bp *SlTCP18*-specific fragments were designed. Hybridization followed the previous description [68] under 45°C conditions.

### Generation of transgenic Physalis plants

CDSs of *POS3-*like were cloned into the plant binary vector *pBAR-A* under the control of the CaMV *35S* promoter to produce transgenic overexpressing *P*. *floridana* plants. A double-stranded RNA interference (RNAi) construct was assembled by introducing a specific 287-bp fragment of *PfPOS3* CDS into the *pFGC1008* vector to generate *PfPOS3*-RNAi plants. For the CRISPR/Cas9-mediated *pfpos3* mutant, two specific 20-bp target sequences in the first exon were chosen to generate an integrated plasmid on the basis of vector *PHSE401* [69]. *Agrobacterium* (strain LBA4404)-mediated *P*. *floridana* transformation was performed following a previous description [24].

### Analysis of morphological traits

The flower radius was measured by the length from the receptacle to the tip of the petal when the flower was fully open. The fruit weight was balanced for each berry. The number of seeds per berry was counted, and 100-seed weight was recorded. The seed size was measured using Image J (National Institutes of Health, Bethesda, Maryland, USA). Ventral surface at the basal region of sepals, ventral surface at tip area of petals, middle region of abaxial surface of anthers, and middle region of dorsal surface of carpels from mature flowers were chosen to measure epidermal cell size using AxioVision (Zeiss, Jena, Germany). For each part of the floral organ, 13–1000 cells were measured. The weight/size of developing berry and the size of epidermal cell during fruit development were quantified in all transgenic *POS3-*like Physalis plants. At each developmental stage, 8–30 developing berries were included, and 159–310 cells were used in evaluating cell size variation using Image J.

### Histological analyses

Tissues were fixed in a formaldehyde–acetic acid–ethanol fix solution (Coolaber, Beijing, China) and dehydrated in an ethanol series. For scanning electron microscope (SEM) analysis, tissues were placed in liquid CO_2_ for drying, coated with gold, and then applied to a scanning electron microscope Hitachi S-4800 (Hitachi, Tokyo, Japan), as described previously [70]. For paraffin sections, the tissues were embedded in Paraplast Plus (Sigma, St. Louis, USA). Sections (10 μm thick) were mounted on slides and stained with safranin-fast green (Solarbio, Beijing, China). Cell size was measured using AxioVision (Zeiss, Jena, Germany).

### Yeast two-hybrid assay

*S. pimpinellifolium* and *S*. *lycopersicum* cDNA libraries were constructed (OEbiotech, Shanghai, China) similar to the Physalis cDNA library [71]. Total RNAs from roots, stems, leaves, floral buds, flowers, and developing fruits were separately isolated and mixed equally. The cDNA libraries were commercially constructed by OE biotech Co., Ltd. (Shanghai, China). The CDSs of the related genes were inserted into the *pGBKT7* and *pGADT7* vectors and transformed into the yeast strain Y2H Gold (Clontech, Mountain View, USA). Yeast cDNA libraries were screened using truncated PfPOS3 and full-length SlPOS3 under appropriate concentrations of 3-amino-1,2,4-triazole (3-AT). The resultant constructs were co-transformed into Y2H Gold, and strains were grown on the selected plates at 28°C for 5 days following the manual protocol of the Matchmaker GAL4 Two-Hybrid System (Clontech, Mountain View, USA).

### Transient expression assay

The CDSs of *POS3-*like, *TCP15*, and *TCP18* were fused to the green fluorescence protein (GFP) in the *pSuper1300* vector [72] driven by the *Super* promoter to construct subcellular localization vectors. The *Super*-GFP empty vector was used as a control. The CDSs of *POS3-*like, *TCP15*, and *TCP18* were cloned into bimolecular fluorescence complementation (BiFC) vectors *pSPYNE-35S* and *pSPYCE-35S* containing the N-terminal and C-terminal half of the yellow fluorescence protein (YFP) [73]. The resultant constructs were transformed into *Agrobacterium tumefaciens* strain GV3101 and infiltrated into the leaves of *N*. *benthamiana* or *P*. *floridana*. The GFP or YFP signals were detected 2 days after injection under an Olympus FV1000MPE confocal laser scanning microscope (Olympus, Japan).

### VIGS analysis

The 285-, 360-, and 219-bp CDSs of *PfPOS3*, *PfTCP15*, and *PfTCP18* were inserted into the *TRV2* vector. The constructed vectors were transformed into *A*. *tumefaciens* strain GV3101 and infiltrated into the leaves of two-week-old seedlings of *P*. *floridana* and *pfpos3* mutants. The conditions and procedures for all manipulations followed a previous description [39].

### Dual-luciferase reporter assay

The reporter plasmids were generated by inserting approximately 1.0 kb promoters of *CYCD1;1* and *CYCB1;1* into *pGreen II-0800-LUC* [74]. The mutant versions were created using site-directed mutagenesis using mutated primers (Table S8). The CDSs of *POS3-*like, *TCP15*, and *TCP18* were inserted into *pJIT163* [75] to construct the effector plasmids. The specifically indicated combination of effector and reporter plasmids was co-transformed into the protoplasts of *P*. *floridana* petals, which were prepared following a previous description [76].

### Chromatin immunoprecipitation-qPCR analysis

*Agrobacterium* injection was performed in 2-week-old *P*. *floridana* seedlings to induce the expression of the pSuper-GFP, PfTCP15-GFP, PfTCP18-GFP, or PfPOS3-GFP fusion proteins. Two days after injection, approximately 3.0 g of leaves were used to extract chromatin and DNA, following a previous study [76]. qPCR was performed using the SYBR® *Premix Ex Taq*™ kit (Takara, Dalian, China) with gene-specific primers (Table S8).

### Primer synthesis and sequencing analysis

The primers used in this work (Table S8) were commercially synthesized, and the isolated sequences and resultant constructs were commercially sequenced by Tsingke Biotechnology Co., Ltd. (Beijing, China).

### Statistical analyses

Statistical analyses, including correlations and significance tests, were performed using SPSS (IBM, NY, USA).

## Supplementary Material

Web_Material_uhaf211

## Data Availability

All data are available in the manuscript or the Supplementary data. All related sequences reported in this work have been deposited in the NCBI GenBank under the accession numbers of OQ248608–OQ248622 and OQ248636–OQ248641.

## References

[1] Borghi L, Bureau M, Simon R. *Arabidopsis JAGGED LATERAL ORGANS* is expressed in boundaries and coordinates *KNOX* and *PIN* activity. Plant Cell. 2007;19:1795–808 17557810 10.1105/tpc.106.047159 PMC1955719

[2] Majer C, Hochholdinger F. Defining the boundaries: structure and function of LOB domain proteins. Trends Plant Sci. 2011;16:47–52 20961800 10.1016/j.tplants.2010.09.009

[3] Kim MJ, Kim M, Lee MR. et al. *LATERAL ORGAN BOUNDARIES DOMAIN* (*LBD*)*10* interacts with *SIDECAR POLLEN/LBD27* to control pollen development in Arabidopsis. Plant J. 2015;81:794–809 25611322 10.1111/tpj.12767

[4] Xu CZ, Luo F, Hochholdinger F. LOB domain proteins: beyond lateral organ boundaries. Trends Plant Sci. 2016;21:159–67 26616195 10.1016/j.tplants.2015.10.010

[5] Xu CY, Cao HF, Zhang QQ. et al. Control of auxin-induced callus formation by bZIP59-LBD complex in *Arabidopsis* regeneration. Nat Plants. 2018;4:108–15 29358751 10.1038/s41477-017-0095-4

[6] Han Z, Yang T, Guo Y. et al. The transcription factor PagLBD3 contributes to the regulation of secondary growth in *Populus*. J Exp Bot. 2021;72:7092–106 34313722 10.1093/jxb/erab351

[7] Omary M, Gil-Yarom N, Yahav C. et al. A conserved superlocus regulates above- and belowground root initiation. Science. 2022;375:eabf4368 35239373 10.1126/science.abf4368

[8] Lee HW, Kim NY, Lee DJ. et al. *LBD18/ASL20* regulates lateral root formation in combination with *LBD16/ASL18* downstream of *ARF7* and *ARF19* in Arabidopsis. Plant Physiol. 2009;151:1377–89 19717544 10.1104/pp.109.143685 PMC2773067

[9] Pandey SK, Lee HW, Kim MJ. et al. LBD18 uses a dual mode of a positive feedback loop to regulate ARF expression and transcriptional activity in Arabidopsis. Plant J. 2018;95:233–51 29681137 10.1111/tpj.13945

[10] Lee HW, Cho C, Pandey SK. et al. *LBD16* and *LBD18* acting downstream of *ARF7* and *ARF19* are involved in adventitious root formation in Arabidopsis. BMC Plant Biol. 2019;19:46 30704405 10.1186/s12870-019-1659-4 PMC6357364

[11] Lee HW, Kim MJ, Kim NY. et al. LBD18 acts as a transcriptional activator that directly binds to the *EXPANSIN14* promoter in promoting lateral root emergence of Arabidopsis. Plant J. 2013;73:212–24 22974309 10.1111/tpj.12013

[12] Shi YN, Vrebalov J, Zheng H. et al. A tomato LATERAL ORGAN BOUNDARIES transcription factor, *SlLOB1*, predominantly regulates cell wall and softening components of ripening. Proc Natl Acad Sci USA. 2021;118:e2102486118 34380735 10.1073/pnas.2102486118 PMC8379924

[13] Dong RR, Yuan YQ, Liu ZQ. et al. *ASYMMETRIC LEAVES 2* and *ASYMMETRIC LEAVES 2-LIKE* are partially redundant genes and essential for fruit development in tomato. Plant J. 2023;114:1285–300 36932869 10.1111/tpj.16193

[14] Liu L, Zhang JL, Xu JY. et al. SlMYC2 promotes SlLBD40-mediated cell expansion in tomato fruit development. Plant J. 2024;118:1872–88 38481350 10.1111/tpj.16715

[15] Knapp S . Tobacco to tomatoes: a phylogenetic perspective on fruit diversity in the Solanaceae. J Exp Bot. 2002;53:2001–22 12324525 10.1093/jxb/erf068

[16] He CY, Münster T, Saedler H. On the origin of floral morphological novelties. FEBS Lett. 2004;567:147–51 15165908 10.1016/j.febslet.2004.02.090

[17] Wang L, Li J, Zhao J. et al. Evolutionary developmental genetics of fruit morphological variation within the Solanaceae. Front Plant Sci. 2015;6:248 25918515 10.3389/fpls.2015.00248 PMC4394660

[18] Grandillo S, Ku HM, Tanksley SD. Identifying the loci responsible for natural variation in fruit size and shape in tomato. Theor Appl Genet. 1999;99:978–87

[19] Frary A, Nesbitt TC, Grandillo S. et al. *fw2.2*: a quantitative trait locus key to the evolution of tomato fruit size. Science. 2000;289:85–8 10884229 10.1126/science.289.5476.85

[20] Muños S, Ranc N, Botton E. et al. Increase in tomato locule number is controlled by two single-nucleotide polymorphisms located near *WUSCHEL*. Plant Physiol. 2011;156:2244–54 21673133 10.1104/pp.111.173997 PMC3149950

[21] Zhang N, Brewer MT, van der Knaap E. Fine mapping of *fw3.2* controlling fruit weight in tomato. Theor Appl Genet. 2012;125:273–84 22406954 10.1007/s00122-012-1832-8

[22] Xu C, Liberatore KL, MacAlister CA. et al. A cascade of arabinosyltransferases controls shoot meristem size in tomato. Nat Genet. 2015;47:784–92 26005869 10.1038/ng.3309

[23] Yuste-Lisbona FJ, Fernández-Lozano A, Pineda B. et al. *ENO* regulates tomato fruit size through the floral meristem development network. Proc Natl Acad Sci USA. 2020;117:8187–95 32179669 10.1073/pnas.1913688117 PMC7148573

[24] He CY, Saedler H. Heterotopic expression of *MPF2* is the key to the evolution of the Chinese lantern of *Physalis*, a morphological novelty in Solanaceae. Proc Natl Acad Sci USA. 2005;102:5779–84 15824316 10.1073/pnas.0501877102 PMC556287

[25] Lu JJ, Luo MF, Wang L. et al. The *Physalis floridana* genome provides insights into the biochemical and morphological evolution of *Physalis* fruits. Hortic Res. 2021;8:244 34795210 10.1038/s41438-021-00705-w PMC8602270

[26] Wang L, He LL, Li J. et al. Regulatory change at *Physalis organ size 1* correlates to natural variation in tomatillo reproductive organ size. Nat Commun. 2014;5:4271 24980282 10.1038/ncomms5271

[27] Wang L, Liu XY, Li QR. et al. A lineage-specific arginine in POS1 is required for fruit size control in Physaleae (Solanaceae) via gene co-option. Plant J. 2022;111:183–204 35481627 10.1111/tpj.15786

[28] Li ZC, He CY. *Physalis floridana cell number Regulator1* encodes a cell membrane-anchored modulator of cell cycle and negatively controls fruit size. J Exp Bot. 2015;66:257–70 25305759 10.1093/jxb/eru415 PMC4265161

[29] Lemmon ZH, Reem NT, Dalrymple J. et al. Rapid improvement of domestication traits in an orphan crop by genome editing. Nat Plants. 2018;4:766–70 30287957 10.1038/s41477-018-0259-x

[30] Gao HH, Li J, Wang L. et al. Transcriptomic variation of the flower–fruit transition in *Physalis* and *Solanum*. Planta. 2020;252:28 32720160 10.1007/s00425-020-03434-x

[31] Parapunova V, Busscher M, Busscher-Lange J. et al. Identification, cloning and characterization of the tomato TCP transcription factor family. BMC Plant Biol. 2014;14:157 24903607 10.1186/1471-2229-14-157 PMC4070083

[32] Li CX, Potuschak T, Colón-Carmona A. et al. *Arabidopsis* TCP20 links regulation of growth and cell division control pathways. Proc Natl Acad Sci USA. 2005;102:12978–83 16123132 10.1073/pnas.0504039102 PMC1200278

[33] Zhang GF, Zhao HT, Zhang CG. et al. TCP7 functions redundantly with several class I TCPs and regulates endoreplication in *Arabidopsis*. J Integr Plant Biol. 2019;61:1151–70 30474211 10.1111/jipb.12749

[34] Husbands A, Bell EM, Shuai B. et al. LATERAL ORGAN BOUNDARIES defines a new family of DNA-binding transcription factors and can interact with specific bHLH proteins. Nucleic Acids Res. 2007;35:6663–71 17913740 10.1093/nar/gkm775 PMC2095788

[35] Shuai B, Reynaga-Peña CG, Springer PS. The *LATERAL ORGAN BOUNDARIES* gene defines a novel, plant-specific gene family. Plant Physiol. 2002;129:747–61 12068116 10.1104/pp.010926 PMC161698

[36] Yang Y, Yu XB, Wu P. Comparison and evolution analysis of two rice subspecies *LATERAL ORGAN BOUNDARIES* domain gene family and their evolutionary characterization from *Arabidopsis*. Mol Phylogenet Evol. 2006;39:248–62 16290186 10.1016/j.ympev.2005.09.016

[37] Wang XF, Liu X, Su L. et al. Identification, evolution and expression analysis of the LBD gene family in tomato. Sci Agric Sin. 2013;46:2501–13

[38] Ba LJ, Shan W, Kuang JF. et al. The banana MaLBD (LATERAL ORGAN BOUNDARIES DOMAIN) transcription factors regulate *EXPANSIN* expression and are involved in fruit ripening. Plant Mol Biol Report. 2014;32:1103–13

[39] Zhang JS, Zhao J, Zhang SH. et al. Efficient gene silencing mediated by tobacco rattle virus in an emerging model plant *Physalis*. PLoS One. 2014;9:e85534 24454885 10.1371/journal.pone.0085534 PMC3891815

[40] Li C, Zhu S, Zhang H. et al. OsLBD37 and OsLBD38, two class II type LBD proteins, are involved in the regulation of heading date by controlling the expression of *Ehd1* in rice. Biochem Biophys Res Commun. 2017;486:720–5 28342864 10.1016/j.bbrc.2017.03.104

[41] Wang H, Han X, Fu X. et al. Overexpression of *TaLBD16-4D* alters plant architecture and heading date in transgenic wheat. Front Plant Sci. 2022;13:911993 36212357 10.3389/fpls.2022.911993 PMC9533090

[42] Tao R, Trivedi I, Trimborn L. et al. TCP3 is a substrate of the COP1/SPA ubiquitin ligase to regulate anthocyanin accumulation and flowering time in *Arabidopsis*. Proc Natl Acad Sci USA. 2025;122:e2426423122 40359052 10.1073/pnas.2426423122 PMC12107181

[43] Zhang L, Wang P, Wang M. et al. GmTCP40 promotes soybean flowering under long-day conditions by binding to the *GmAP1a* promoter and upregulating its expression. Biomolecules. 2024;14:465 38672481 10.3390/biom14040465 PMC11047976

[44] Zhang J, Tang W, Huang Y. et al. Down-regulation of a *LBD*-like gene, *OsIG1*, leads to occurrence of unusual double ovules and developmental abnormalities of various floral organs and megagametophyte in rice. J Exp Bot. 2015;66:99–112 25324400 10.1093/jxb/eru396 PMC4265153

[45] Rast MI, Simon R. *Arabidopsis JAGGED LATERAL ORGANS* acts with *ASYMMETRIC LEAVES2* to coordinate *KNOX* and *PIN* expression in shoot and root meristems. Plant Cell. 2012;24:2917–33 22822207 10.1105/tpc.112.099978 PMC3426123

[46] Lan J, Wang N, Wang Y. et al. Arabidopsis TCP4 transcription factor inhibits high temperature-induced homeotic conversion of ovules. Nat Commun. 2023;14:5673 37704599 10.1038/s41467-023-41416-1 PMC10499876

[47] Huang T, Irish VF. Temporal control of plant organ growth by TCP transcription factors. Curr Biol. 2015;25:1765–70 26073137 10.1016/j.cub.2015.05.024

[48] Li M, Nie C, He S. et al. VvARF19 represses VvLBD13-mediated cell wall degradation to delay softening of grape berries. Hortic Res. 2024;12:uhae322 40041604 10.1093/hr/uhae322 PMC11879436

[49] Song CB, Shan W, Yang YY. et al. Heterodimerization of MaTCP proteins modulates the transcription of *MaXTH10/11* genes during banana fruit ripening. Biochim Biophys Acta Gene Regul Mech. 2018;1861:613–22 29935343 10.1016/j.bbagrm.2018.06.005

[50] An JP, Liu YJ, Zhang XW. et al. Dynamic regulation of anthocyanin biosynthesis at different light intensities by the BT2-TCP46-MYB1 module in apple. J Exp Bot. 2020;71:3094–109 31996900 10.1093/jxb/eraa056 PMC7475178

[51] Zhang Y, Chen C, Cui Y. et al. Potential regulatory genes of light induced anthocyanin accumulation in sweet cherry identified by combining transcriptome and metabolome analysis. Front Plant Sci. 2023;14:1238624 37662172 10.3389/fpls.2023.1238624 PMC10469515

[52] Martín-Trillo M, Cubas P. TCP genes: a family snapshot ten years later. Trends Plant Sci. 2010;15:31–9 19963426 10.1016/j.tplants.2009.11.003

[53] Li ZY, Li B, Shen WH. et al. TCP transcription factors interact with AS2 in the repression of class-I *KNOX* genes in *Arabidopsis thaliana*. Plant J. 2012;71:99–107 22380849 10.1111/j.1365-313X.2012.04973.x

[54] Cubas P, Lauter N, Doebley J. et al. The TCP domain: a motif found in proteins regulating plant growth and development. Plant J. 1999;18:215–22 10363373 10.1046/j.1365-313x.1999.00444.x

[55] Qi FF, Zhang FX. Cell cycle regulation in the plant response to stress. Front Plant Sci. 2020;10:1765 32082337 10.3389/fpls.2019.01765 PMC7002440

[56] Jing W, Gong F, Liu G. et al. Petal size is controlled by the MYB73/TPL/HDA19-miR159-CKX6 module regulating cytokinin catabolism in *Rosa hybrida*. Nat Commun. 2023;14:7106 37925502 10.1038/s41467-023-42914-y PMC10625627

[57] Zhang F, Wang J, Ding T. et al. MYB2 and MYB108 regulate lateral root development by interacting with LBD29 in *Arabidopsis thaliana*. J Integr Plant Biol. 2024;66:1675–87 38923126 10.1111/jipb.13720

[58] Dong J, Wang Y, Xu L. et al. *RsLBD3* regulates the secondary growth of taproot by integrating auxin and cytokinin signaling in radish (*Raphanus sativus* L.). J Integr Plant Biol. 2025;67:1823–42 40331562 10.1111/jipb.13918

[59] Davière JM, Wild M, Regnault T. et al. Class I TCP-DELLA interactions in inflorescence shoot apex determine plant height. Curr Biol. 2014;24:1923–8 25127215 10.1016/j.cub.2014.07.012

[60] Grant PR, Grant BR. Unpredictable evolution in a 30-year study of Darwin’s finches. Science. 2002;296:707–11 11976447 10.1126/science.1070315

[61] Wu P, Jiang TX, Suksaweang S. et al. Molecular shaping of the beak. Science. 2004;305:1465–6 15353803 10.1126/science.1098109 PMC4380220

[62] Eriksson O, Friis EM, Löfgren P. Seed size, fruit size, and dispersal systems in angiosperms from the early cretaceous to the late tertiary. Am Nat. 2000;156:47–58 10824020 10.1086/303367

[63] Schmittgen TD, Livak KJ. Analyzing real-time PCR data by the comparative *C*_T_ method. Nat Protoc. 2008;3:1101–8 18546601 10.1038/nprot.2008.73

[64] Kumar S, Stecher G, Li M. et al. MEGA X: molecular evolutionary genetics analysis across computing platforms. Mol Biol Evol. 2018;35:1547–9 29722887 10.1093/molbev/msy096 PMC5967553

[65] Nguyen LT, Schmidt HA, von Haeseler A. et al. IQ-TREE: a fast and effective stochastic algorithm for estimating maximum-likelihood phylogenies. Mol Biol Evol. 2015;32:268–74 25371430 10.1093/molbev/msu300 PMC4271533

[66] Tang HB, Bowers JE, Wang XY. et al. Synteny and collinearity in plant genomes. Science. 2008;320:486–8 18436778 10.1126/science.1153917

[67] Brewer PB, Heisler MG, Hejátko J. et al. *In situ* hybridization for mRNA detection in *Arabidopsis* tissue sections. Nat Protoc. 2006;1:1462–7 17406436 10.1038/nprot.2006.226

[68] Xu YY, Chong K, Xu ZH. et al. The practical technique of *in situ* hybridization with RNA probe. Chin Bull Bot. 2002;19:234–8

[69] Xing HL, Dong L, Wang ZP. et al. A CRISPR/Cas9 toolkit for multiplex genome editing in plants. BMC Plant Biol. 2014;14:327 25432517 10.1186/s12870-014-0327-y PMC4262988

[70] Chen CB, Wang SP, Huang H. *LEUNIG* has multiple functions in gynoecium development in *Arabidopsis*. Genesis. 2000;26:42–54 10660672 10.1002/(sici)1526-968x(200001)26:1<42::aid-gene7>3.0.co;2-j

[71] Zhao J, Gong PC, Liu HY. et al. Multiple and integrated functions of floral C-class MADS-box genes in flower and fruit development of *Physalis floridana*. Plant Mol Biol. 2021;107:101–16 34424500 10.1007/s11103-021-01182-4

[72] Chen YF, Li LQ, Xu Q. et al. The WRKY6 transcription factor modulates *PHOSPHATE1* expression in response to low pi stress in *Arabidopsis*. Plant Cell. 2009;21:3554–66 19934380 10.1105/tpc.108.064980 PMC2798333

[73] Walter M, Chaban C, Schütze K. et al. Visualization of protein interactions in living plant cells using bimolecular fluorescence complementation. Plant J. 2004;40:428–38 15469500 10.1111/j.1365-313X.2004.02219.x

[74] Hellens RP, Allan AC, Friel EN. et al. Transient expression vectors for functional genomics, quantification of promoter activity and RNA silencing in plants. Plant Mothods. 2005;1:13 10.1186/1746-4811-1-13 PMC1334188 16359558

[75] Guerineau F, Lucy A, Mullineaux P. Effect of two consensus sequences preceding the translation initiator codon on gene expression in plant protoplasts. Plant Mol Biol. 1992;18:815–8 1373083 10.1007/BF00020027

[76] Gong PC, Song CJ, Liu HY. et al. *Physalis floridana CRABS CLAW* mediates neofunctionalization of *GLOBOSA* genes in carpel development. J Exp Bot. 2021;72:6882–903 34181715 10.1093/jxb/erab309 PMC8547157

