# Supplementary material for: Heterometric expression of an LBD gene via LBD-TCP assembly regulates floral organ size and fruit weight in Physalis
Source: Hortic Res. 2025 Aug 11;12(11):uhaf211. doi: 10.1093/hr/uhaf211 (PMC12578467; doi:10.1093/hr/uhaf211)
Supplement: Web_Material_uhaf211 [file web_material_uhaf211.zip › HR-2025-456-supplFigures-R1.pdf]

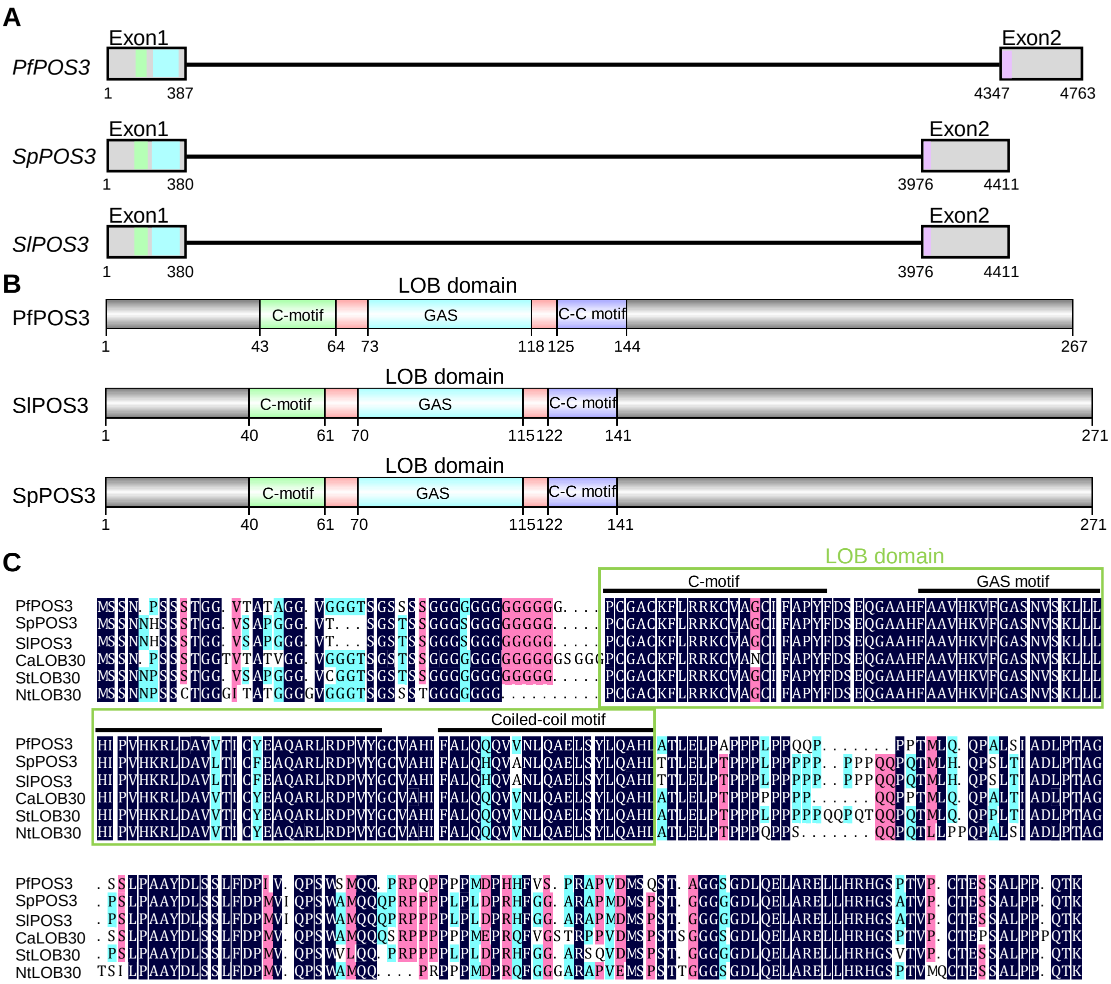

Figure S1 Gene structure and putative protein sequence of *POS3-like*.
(**A**) The exon-intron organization of *POS3-like*. The exons and introns are respectively represented by the gray boxes and black lines. The colored parts represent the composition of LOB domain. The numbers indicate the positions of exons. (**B**) The protein structure of POS3-like. The colored boxes represent the substructures in the LOB domain as indicated, and the gray boxes are variable regions. The length of each substructure is indicated by indicated numbers. (**C**) Multiple sequence alignment of POS3-like. The conserved LOB domain is boxed in green, and substructure includes the C motif, GAS motif, and Coiled-coil motif corresponding to **B**. The black ground represents the conserved amino acid residues, and the pink, cyan, and white backgrounds respectively represent identities ranged from 75–99%, 50–75%, and 0–50% in amino acid sequences. Ca, *Capsicum annuum*; St, *Solanum tuberosum*; Nt, *Nicotiana tabacum*.

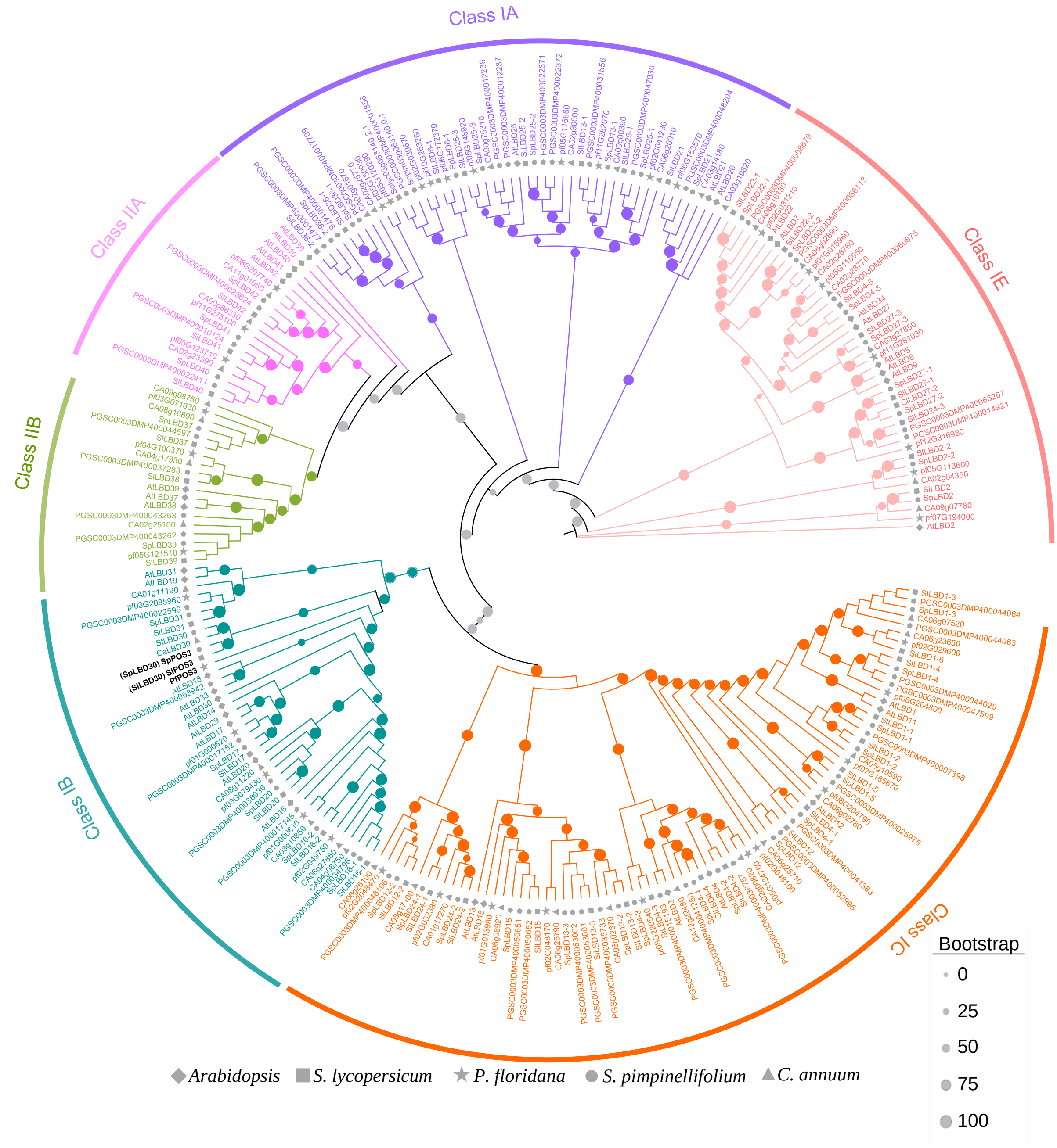

Figure S2 Phylogenetic analysis of the LBD family.
The LBD genes were genome-wide surveyed from *Arabidopsis*, *S. lycopersicum*, *S. pimpinellifolium*, *S. tuberosum*, *C. annuum*, and *P. floridana* (Table S2). The maximum likelihood (ML) unrooted tree was constructed, and the bootstrap value is proportionally indicated with the dot. LBD genes were divided into Class I and Class II. The POS3-like is highlighted in black bold. Different symbols represent different species.

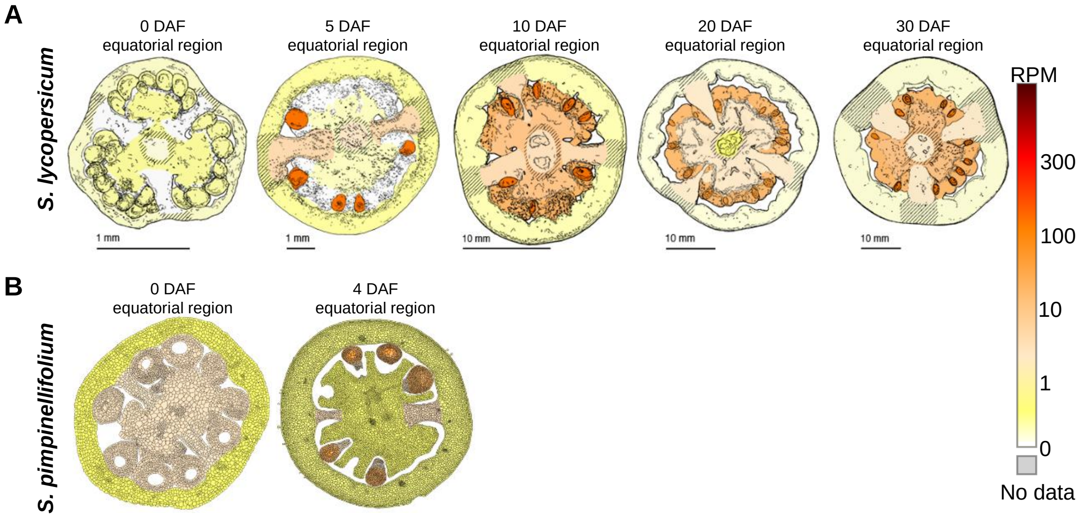

Figure S3 Expression patterns of *POS3-like* genes in solanaceous species.
(A) Expression pattern of *SlPOS3* in *S. lycopersicum* M82 during fruit development. (**B**) Expression pattern of *SpPOS3* in *S. pimpinellifolium* during fruit development. The data in **A** and **B** were collected from Tomato Expression Atlas (https://tea.solgenomics.net/). DAF, days after fertilization; RPM, reads per million mapped reads.

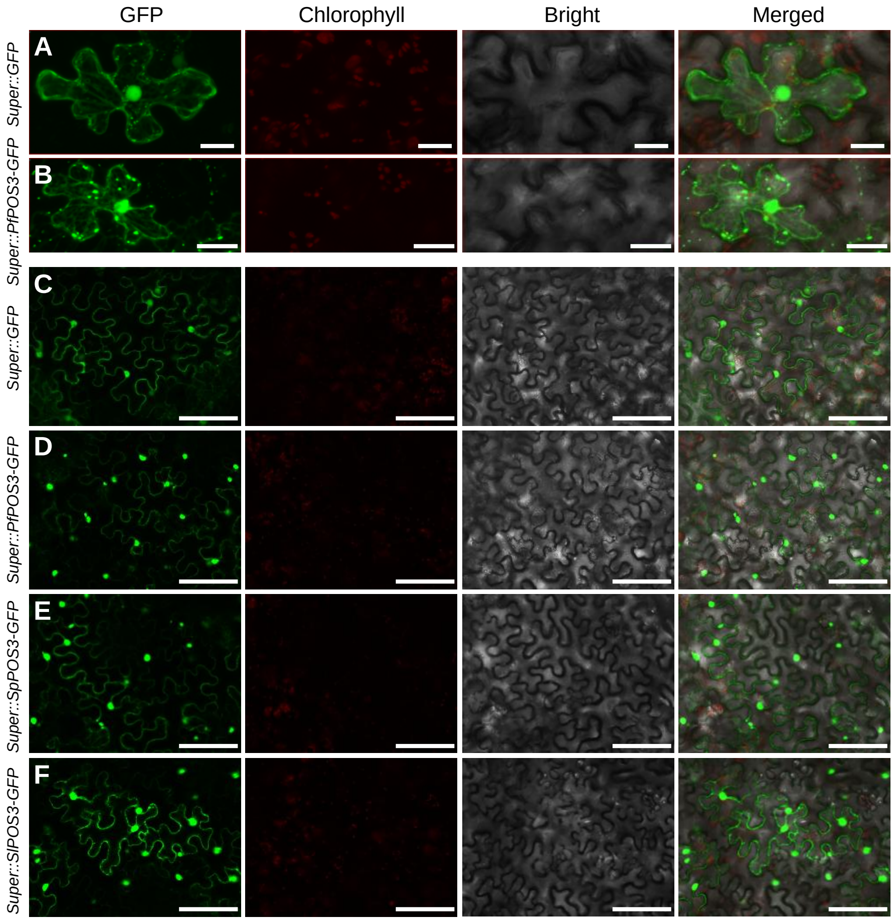

Figure S4 Subcellular localization of POS3-like.
(**A**) The GFP control. (**B**) PfPOS3-GFP fusion proteins. The indicated constructs in **A** and **B** were transformed into epidermal cells of *P. floridana* leaves. Bars = 20 μm. (**C**) The GFP control. (**D**) PfPOS3-GFP fusion proteins. (**E**) SpPOS3-GFP fusion proteins. (**F**) SlPOS3-GFP fusion proteins. The indicated constructs in **C**–**F** were transformed into epidermal cells of tobacco leaves. Bars = 100 μm.

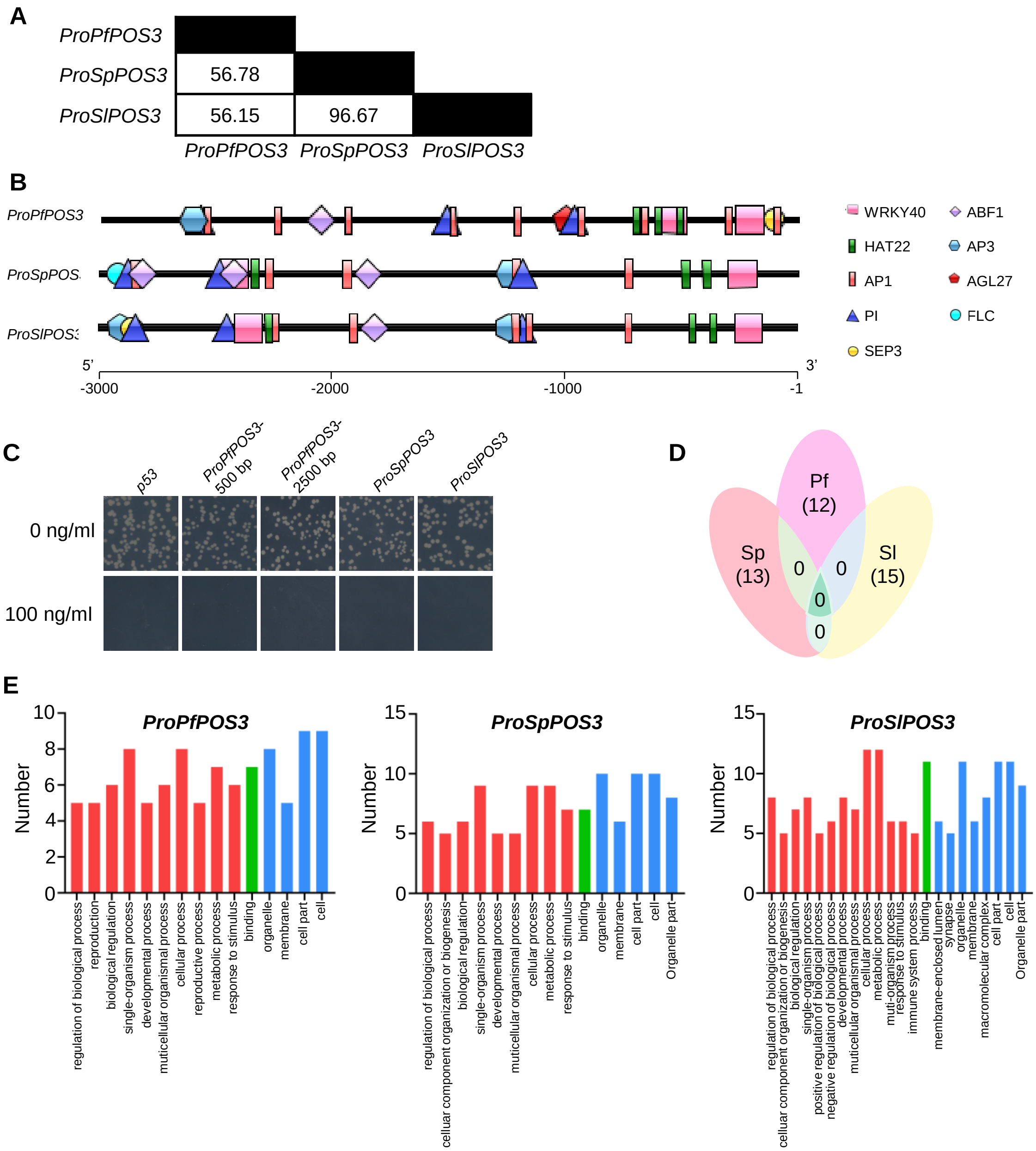

Figure S5 Comparison of *POS3-like* promoters and yeast one-hybrid (Y1H) analysis. (**A**) The sequence identity of the putative promoters of *POS3-like*. *ProPfPOS3*, promoter of *P. floridana POS3*; *ProSpPOS3*, promoter of *S. pimpinellifolium POS3*; *ProSlPOS3*, promoter of *S. lycopersicum POS3*. (**B**) Potential transcription factor (TF) binding sites in *POS3-like* promoters from three species. The predicated TF binding sites are highlighted in rectangle. The divergence in number and distribution of the predicted *cis*-motifs in the *POS3-like* promoters might relate to the heterometric expression of the *POS3* orthologous genes in the three species. (**C**) Determining the minimal inhibitory concentration of Aureobasidin A (AbA) for *POS3-like* promoters. One hundred ng/ml AbA was used in the Y1H library screens. The *p53-AbAi* vector was used as a positive control. (**D**) Venn diagram of the results of library screens using the promoters of *POS3-like*. The number represents putative *trans*-acting factors of each *POS3-like* promoter revealed in library screens (Table S3). About 3.0 kb promoters of *PfPOS3*, *SpPOS3*, and *SlPOS3* were inserted into vector *pAbAi*, and yeast cDNA libraries were screened according to the Yeast One-Hybrid Library Screening System User Manual (Clontech, Mountain View, USA). Pf, *ProPfPOS3*; Sp, *ProSpPOS3*; Sl, *ProSlPOS3*. (**E**) Gene Ontology (GO) analysis of the putative *trans*-acting factors of the indicated *POS3-like* promoters using agriGO (http://systemsbiology.cau.edu.cn/agriGOv2/).

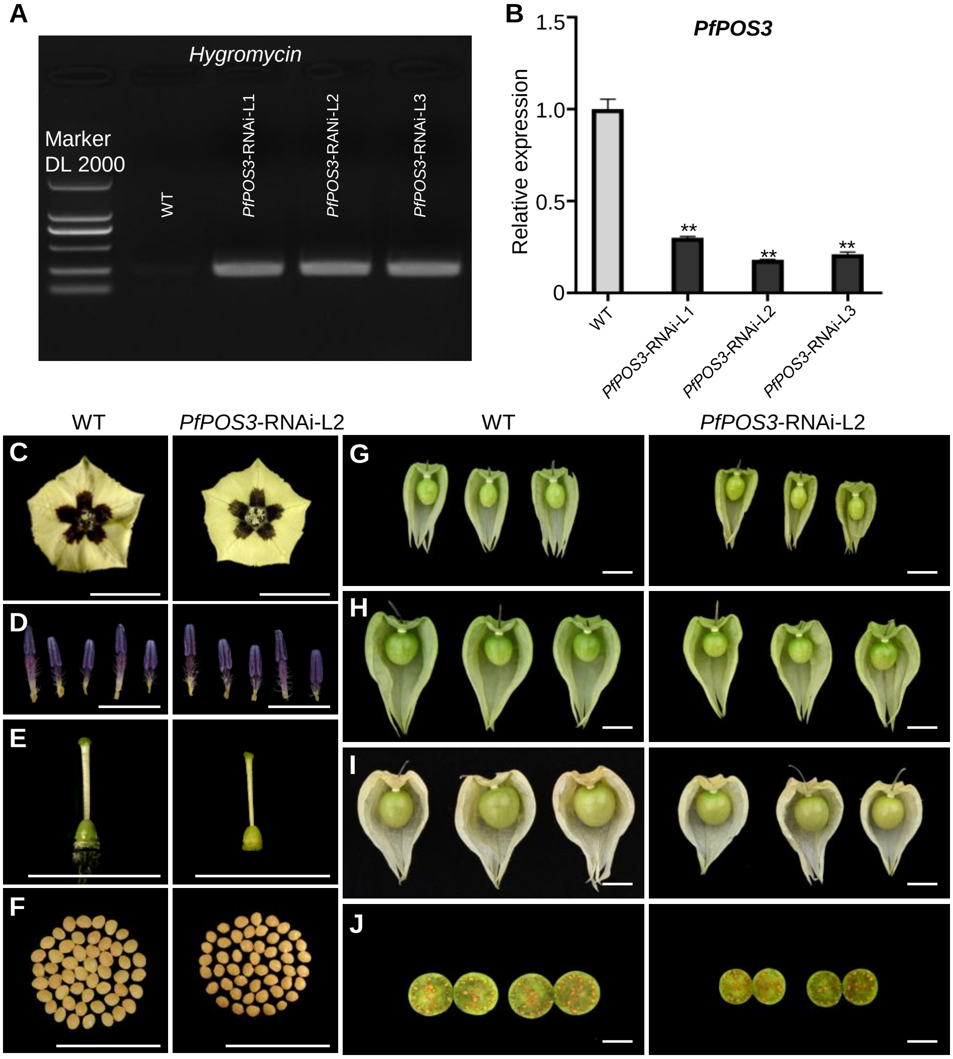

Figure S6 Genotypic and phenotypic analysis of transgenic *PfPOS3*-RNAi *Physalis* plants. (**A**) Identity of transgenic *PfPOS3*-RNAi plants. Amplification of the *Hygromycin* (*Hyg*) gene from the genomic DNA of leaves confirmed the identity of the three indicated transgenic *P. floridana* plant lines (*PfPOS3*-RNAi-L1, *PfPOS3*-RNAi-L2, and *PfPOS3*-RNAi-L3). WT, *P. floridana*. (**B**) The relative expression of *PfPOS3* in *PfPOS3*-RNAi. *PfActin* was used as an internal control. The mean and S.D. are presented (n = 3). *, $P < 0.05$; **, $P < 0.01$ relative to WT in two-tailed Student's *t*-test. (**C**) Flowers of the WT and *PfPOS3*-RNAi-L2. (**D**) Stamens of the WT and *PfPOS3*-RNAi-L2. (**E**) Carpels of the WT and *PfPOS3*-RNAi-L2. (**F**) Seeds of the WT and *PfPOS3*-RNAi-L2. (**G**) Fruit morphology at 10 DAF of the WT and *PfPOS3*-RNAi-L2. (**H**) Fruit morphology at 20 DAF of the WT and *PfPOS3*-RNAi-L2. (**I**) Mature fruits of the WT and *PfPOS3*-RNAi-L2. The lantern was partially removed to show the berry inside in **G**–**I**. (**J**) Mature berries of the WT and *PfPOS3*-RNAi-L2. The berry was cut to show the seeds inside. Bars = 1 cm.

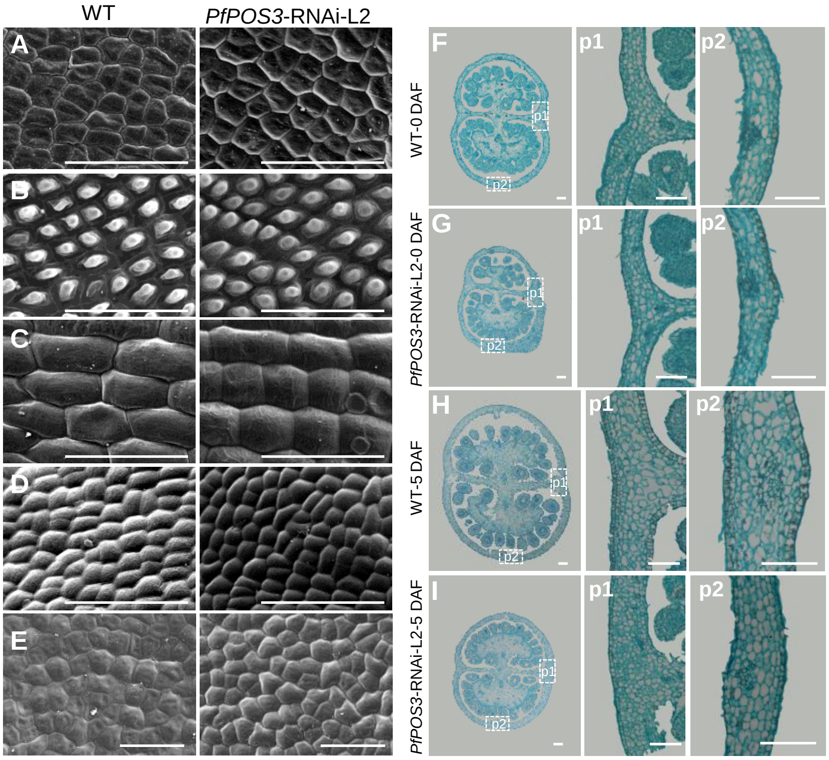

Figure S7 Cytological analysis of floral organs and developing berries of *PfPOS3*-RNAi.

(**A**) Adaxial epidermal cells at the basal region of sepals of the WT and *PfPOS3*-RNAi-L2 revealed by SEM. (**B**) Adaxial epidermal cells at tip area of petals of the WT and *PfPOS3*-RNAi-L2 revealed by SEM. (**C**) Epidermal cells at middle region of abaxial surface of anthers of the WT and *PfPOS3*-RNAi-L2 revealed by SEM. (**D**) Epidermal cells at middle region of dorsal surface of carpels of the WT and *PfPOS3*-RNAi-L2 by SEM. (**E**) Epidermal cells of mature berries of the WT and *PfPOS3*-RNAi-L2. (**F**) Paraffin sections of berries 0 DAF of the WT. (**G**) Paraffin sections of berries 0 DAF of *PfPOS3*-RNAi-L2. (**H**) Paraffin sections of berries 5 DAF of the WT. (**I**)Paraffin sections of berries 5 DAF of *PfPOS3*-RNAi-L2. p1,p2 indicates the positions for cell analysis. Bars = 100 μm.

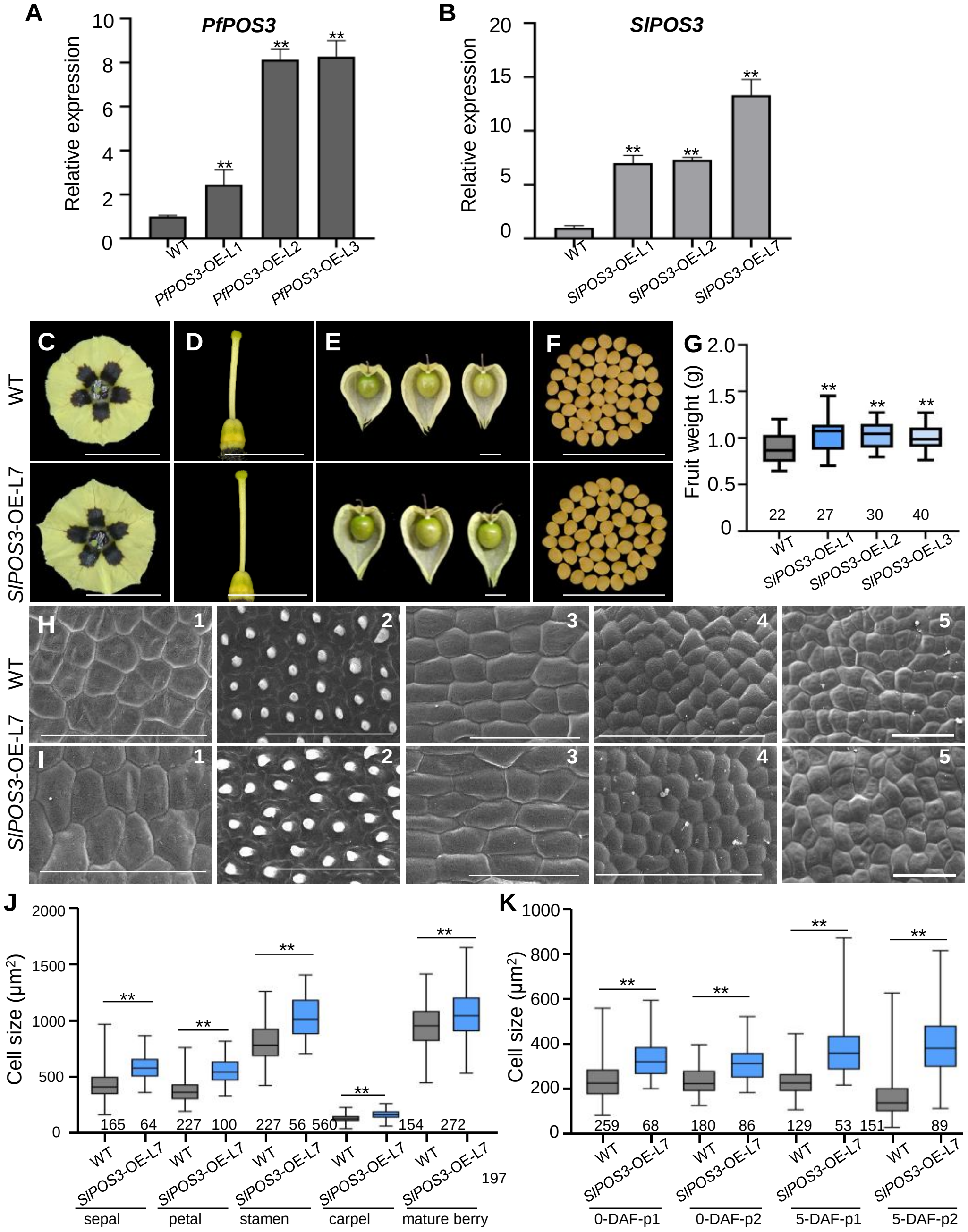

Figure S8 Genotypic and phenotypic analysis of transgenic *POS3*-OE *Physalis* plants (**A**) Transgenic *PfPOS3*-OE plants. The relative expression of *PfPOS3* in *PfPOS3*-OE. *PfActin* was used as an internal control. The mean and S.D. are presented (n = 3). **, $P < 0.01$ relative to WT in two-tailed Student's *t*-test. (**B**)Transgenic *SlPOS3*-OE plants. The relative expression of *SlPOS3* in *SlPOS3*-OE. *PfActin* was used as an internal control. The mean and S.D. are presented (n = 3). **, $P < 0.01$ relative to WT in two-tailed Student's *t*-test. (**C**–**F**) Flowers, carpels, mature fruits, and seeds of the WT and *SlPOS3*-OE-L7. Bars = 1 cm. (**G**) Fruit weight variation of the WT and the three *SlPOS3*-OE lines. (**H, I**) Epidermal cells of floral organs and mature berries of the WT (H) and *SlPOS3*-OE-L7 (I). 1–5 represent the sepal (adaxial surface at the basal region), petal (adaxial surface at tip area), stamen (middle region of abaxial surface), carpel (middle region of dorsal surface) from mature flowers, and mature berries, respectively. Bars = 100 μm. (**J**) Cell size variation of four whorl floral organs and mature berries. (**K**) Cell size variation around p1 and p2 (defined in Figure 2O) of berries 0 DAF and 5 DAF by paraffin sections. In **G**, **J**, and **K**, the mean and S.D. are presented. Sample size was given below each column. *, $P < 0.05$; **, $P < 0.01$ in two-tailed Student's *t*-test.

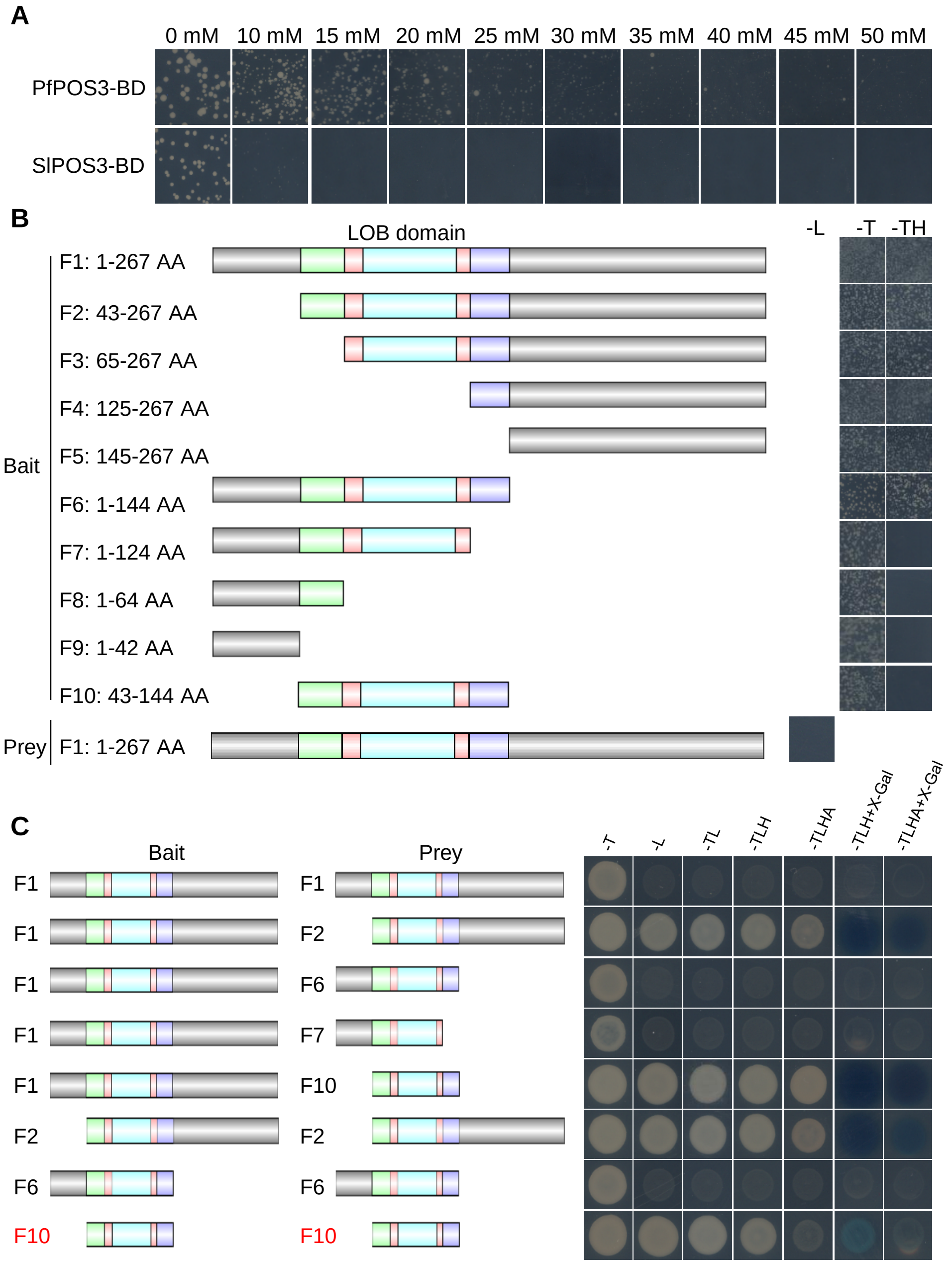

Figure S9 Investigation of self-activation activity of POS3-like.
(**A**) Self-activation test in yeast. The CDS of PfPOS3 and SlPOS3 were integrated to the *pGBKT7* vector (PfPOS3-BD and SlPOS3-BD) and the constructs were transformed into Y2H Gold, which were plated on the synthetically defined medium (SD)/-Trp-His containing different concentrations of 3-AT. PfPOS3 had a strong auto-activation activity, while the weak autoactivation of SlPOS3 was completely repressed by adding 10 mM/L 3-AT. (**B**) Determination of self-activation region and toxicity of PfPOS3. Different regions (F1–F10) were tested and F7–F10 as baits showed no self-activation, while F1 as prey showed toxicity. (**C**) Detection of dimer formation by combining different regions. It turned out that only the combination of F10+F10 (highlighted in red) did not affect dimer formation. Therefore, F10 was used for the subsequent library screens by Y2H. -T, SD/-Trp; -L, SD/-Leu; -TL, SD/-Trp-Leu; -TLH, SD/-Trp-Leu-His; -TLHA, SD/-Trp-Leu-His-Ade; -TLH+X-Gal, SD/-Trp-Leu-His+X-Gal; -TLHA+X-Gal, SD/-Trp-Leu-His-Ade+X-Gal.

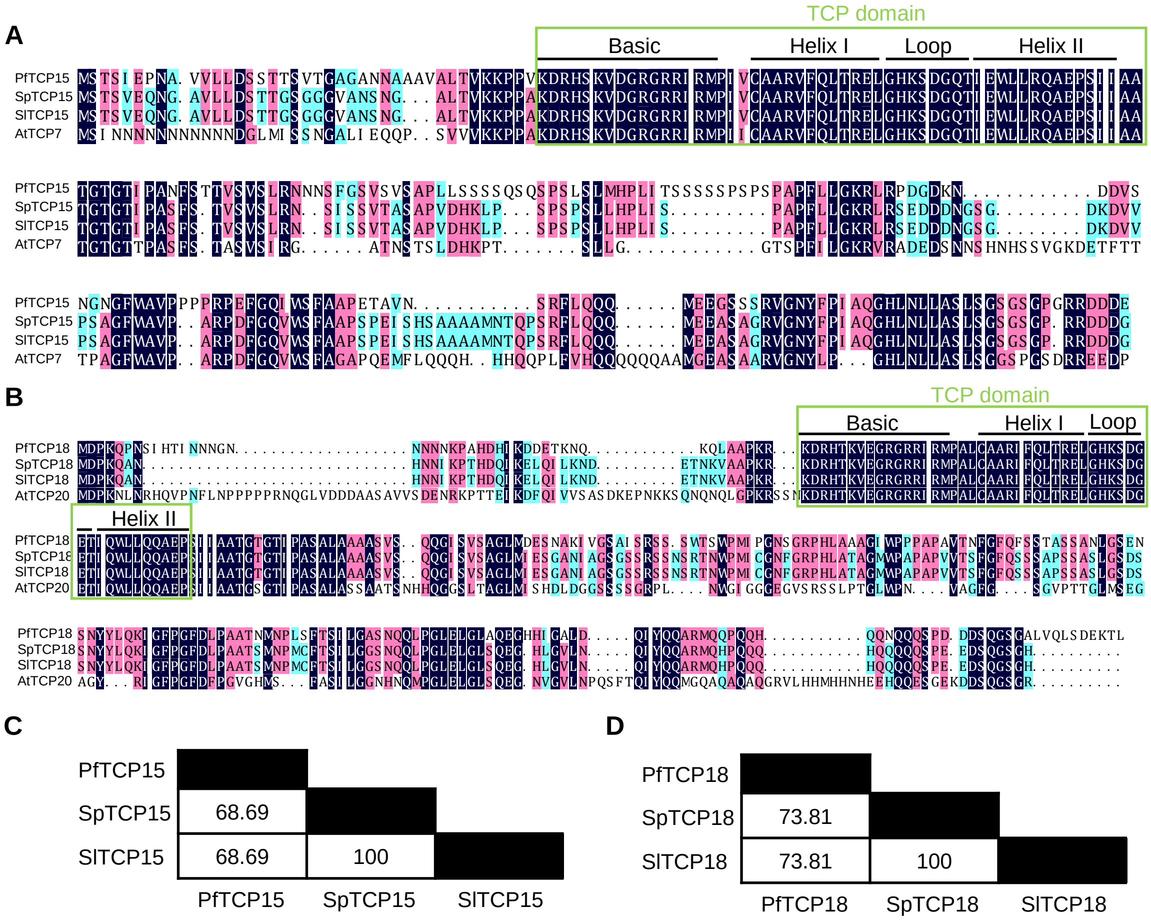

Figure S10 Molecular identification of TCPs from the three indicated solanaceous species. (**A**) Multiple sequence alignment of TCP15 orthologs. (**B**) Multiple sequence alignment of TCP18 orthologs. The conserved TCP domain is indicated, which contains Basic, Helix I, Loop, and Helix II. The black ground represents the conserved amino acid residues, and the pink, cyan and white backgrounds respectively represent identities ranged from 75%–99%, 50%–75%, and 0%–50% in amino acid sequences. (**C**) Sequence identity of TCP15 orthologs. (**D**) Sequence identity of TCP18 orthologs.

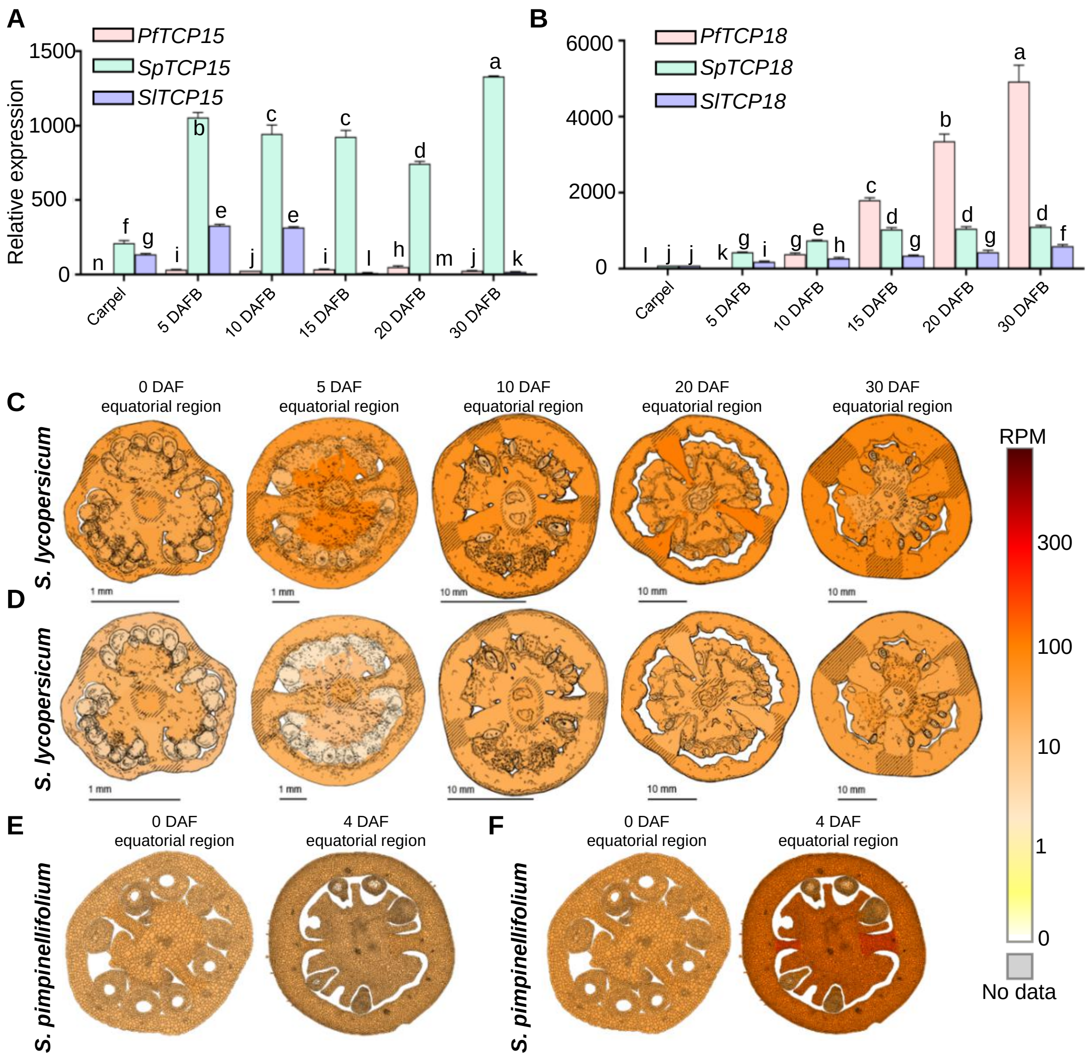

Figure S11 Expression patterns of *TCP15* and *TCP18* in the three solanaceous species. (**A**) Tissue-specific mRNA accumulation of *TCP15* in the three species. (**B**) Tissue-specific mRNA accumulation of *TCP18* in the three species. In **A** and **B**, total RNA from the indicated tissues/organs was subjected to qRT-PCR analysis. The expression of each *TCP* in carpel was set as 1.0. The mean and S.D. are presented (n=3). Different lowercase letters represent significant difference ($P < 0.05$) in two-tailed Student's *t*-test. *PfActin*, *SpActin* or *SlActin* was used as a loading and internal control. DAFB, berry of days after fertilization. (**C**) Expression pattern of *SlTCP15* in *S. lycopersicum* M82 during fruit development. (**D**) Expression pattern of *SlTCP18* in *S. lycopersicum* M82 during fruit development. (**E**) Expression pattern of *SpTCP15* in *S. pimpinellifolium* during fruit development. (**F**) Expression pattern of *SpTCP18* in *S. pimpinellifolium* during fruit development. The data in **C**–**F** were collected from Tomato Expression Atlas (https://tea.solgenomics.net/). DAF, days after fertilization; RPM, reads per million mapped reads.

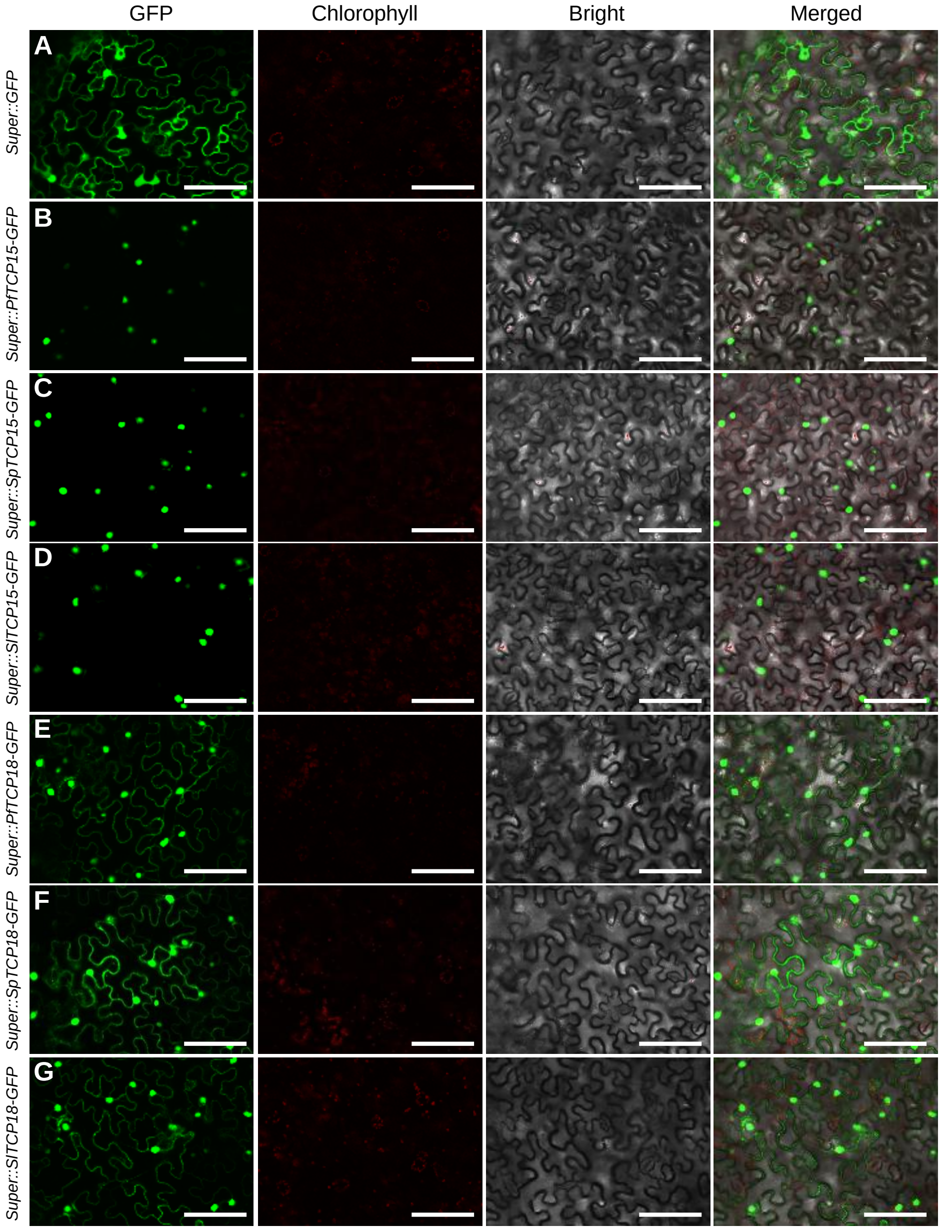

Figure S12 Subcellular localization of TCP15 and TCP18.
(**A**) The GFP control. (**B**) PfTCP15-GFP fusion proteins. (**C**) SpTCP15-GFP fusion proteins. (**D**) SlTCP15-GFP fusion proteins. (**E**) PfTCP18-GFP fusion proteins. (**F**) SpTCP18-GFP fusion proteins. (**G**) SlTCP18-GFP fusion proteins. The indicated constructs were transformed into the epidermal cells of tobacco leaves. Bars = 100 μm.

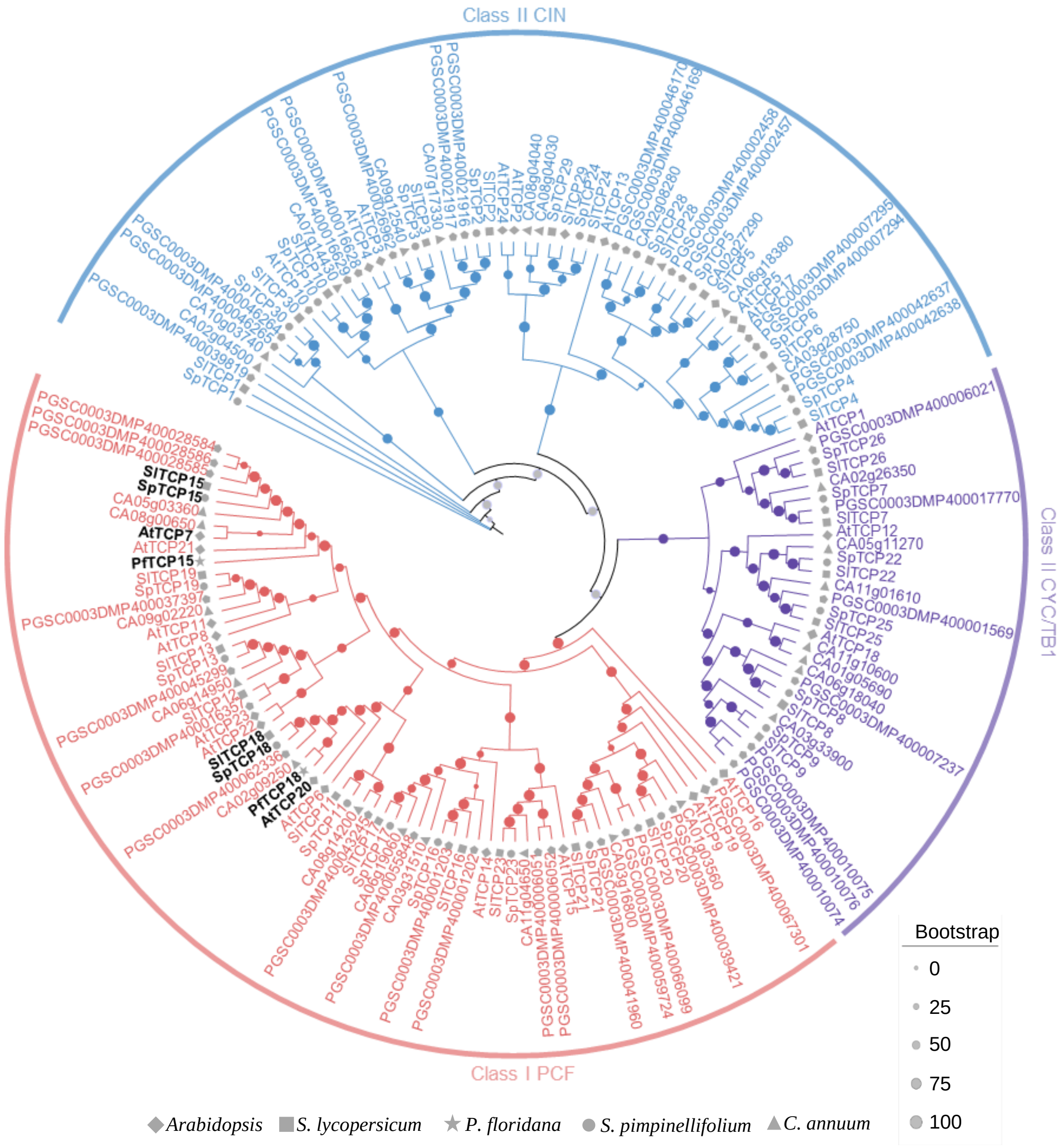

Figure S13 Phylogenetic analysis of the TCP family.
The TCP genes were genome-wide surveyed from *Arabidopsis*, *S. lycopersicum*, *S. pimpinellifolium*, *S. tuberosum*, and *C. annuum* (Table S2). The maximum likelihood (ML) unrooted tree was constructed, and the bootstrap value is proportionally indicated with the dot. TCP genes were divided into Class I and Class II. PCF, Proliferating cell factor; CYC/TB1, Cycloidea/Teosinte Branched 1; CIN, Cincinnata. TCP15 and TCP18 are highlighted in black bold. Different symbols represent different species.

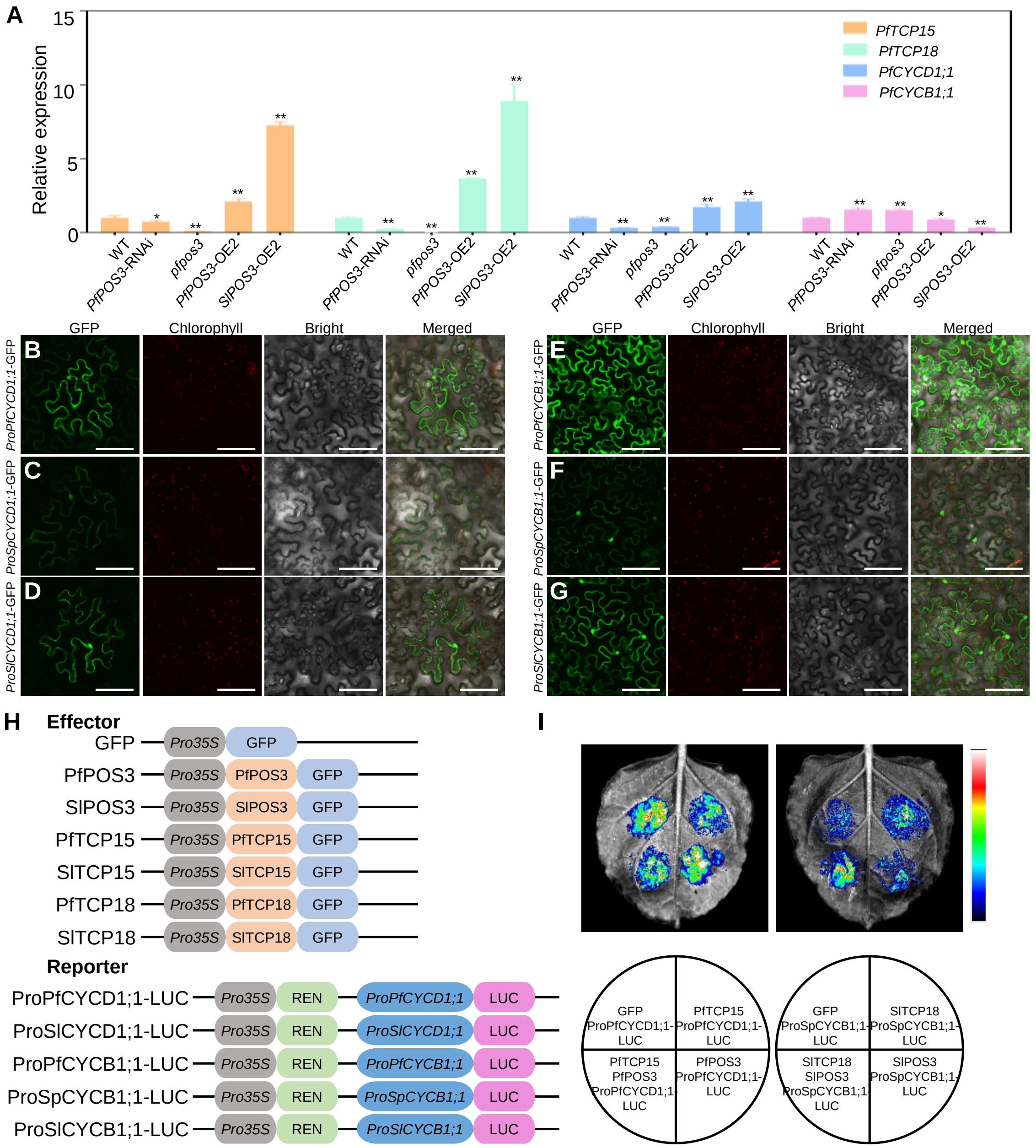

Figure S14 Activity detection of the promoters of *CYCD1;1* and *CYCB1;1*, potential downstream genes of *POS3-like* in the cell cycle.

(**A**) Expression of *PfTCP15*, *PfTCP18*, *PfCYCB1;1*, and *PfCYCD1;1* in the indicated *POS3-like* transgenic *Physalis* plants. To evaluate the variation in gene expression, total RNA from the fruit 5 DAF of the indicated transgenic plants was subjected to qRT-PCR analyses. The *PfActin* gene was used as an internal control. The mean and S.D. are presented (n=3). *, $P < 0.05$; **, $P < 0.01$ in two-tailed Student's *t*-test. (**B**) The activity detection of promoter of *PfCYCD1;1* (*ProPfCYCD1;1*). (**C**) The activity detection of promoter of *SpCYCD1;1* (*ProSpCYCD1;1*). (**D**) The activity detection of promoter of *SlCYCD1;1* (*ProSlCYCD1;1*). (**E**) The activity detection of promoter of *PfCYCB1;1* (*ProPfCYCB1;1*). (**F**) The activity detection of promoter of *SpCYCB1;1* (*ProSpCYCB1;1*). (**G**) The activity detection of promoter of *SlCYCB1;1* (*ProSlCYCB1;1*). In **B**–**G**, the *super* promoter on *pSuper1300* was replaced by *CYCD1;1* or *CYCB1;1* promoter. Bar = 100 μm. (**H**) Schematic diagram of the constructs used in transient expression assay. (**I**) Transient expression of LUC. The *ProPfCYCD1;1-LUC* and *ProSpCYCB1;1-LUC* reporter constructs were transiently infiltrated in tobacco leaves together with the different effectors. The pseudocolor bar shows luminescence intensity. GFP effector was used as a control. It should be noted that the 1.0 Kb putative promoters of *SpCYCD1;1* and *SlCYCD1;1* were identical in sequences, and the *S. lycopersicum* sequences were thus used.

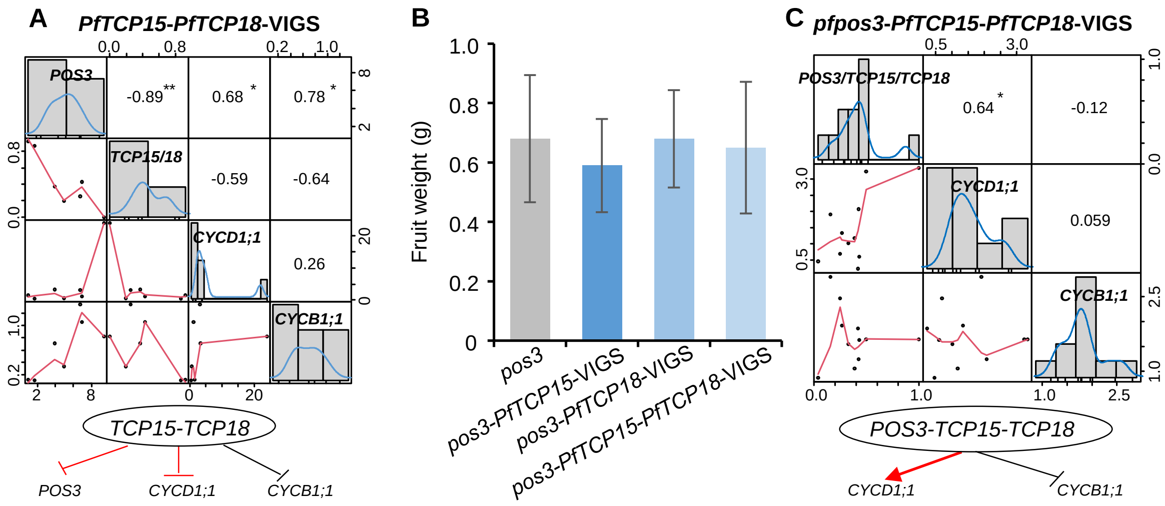

Figure S15 Correlation of the investigated gene expression and traits in virus-induced gene silencing (VIGS) treatments.

(**A**) *PfTCP15-PfTCP18*-VIGS in *P. floridana*. (**B**) Fruit weight variation of the VIGS treatments (*TCP15, TCP18*) in the *pfpos3*. (**C**) *pfpos3-PfTCP15-PfTCP18*-VIGS. In A and C, the distribution of each variable is shown on the diagonal, and the bivariate scatter plots with a fitted line are displayed. The deduced gene regulations are given below each matrix. The arrow indicates a positive correlation, and the T line indicates a negative correlation. The red indicate *P*-value < 0.05; the black indicates *P*-value > 0.05. The Pearson correlation coefficient was calculated in SPSS, and the correlation matrix was visualized using PerformanceAnalytics (https://CRAN.R-project.org/package=PerformanceAnalytics). *, *P* < 0.05; **, *P* < 0.01 in two-tailed Student's *t*-test.
